# Prophase-Specific Perinuclear Actin Coordinates Centrosome Separation and Positioning to Ensure Accurate Chromosome Segregation

**DOI:** 10.1016/j.celrep.2020.107681

**Published:** 2020-05-26

**Authors:** Tom Stiff, Fabio R. Echegaray-Iturra, Harry J. Pink, Alex Herbert, Constantino Carlos Reyes-Aldasoro, Helfrid Hochegger

**Affiliations:** 1 Genome Damage and Stability Centre, School of Life Sciences, University of Sussex, Brighton BN19RQ, UK; 2 GiCentre, Department of Computer Science, City, University of London, London EC1V 0HB, UK

**Keywords:** centrosome separation, Eg5, LINC complex, FHOD1, perinuclear actin, microtubules, centrosome positioning, G2/M transition, mitotic entry, centrosome tracking

## Abstract

Centrosome separation in late G2/ early prophase requires precise spatial coordination that is determined by a balance of forces promoting and antagonizing separation. The major effector of centrosome separation is the kinesin Eg5. However, the identity and regulation of Eg5-antagonizing forces is less well characterized. By manipulating candidate components, we find that centrosome separation is reversible and that separated centrosomes congress toward a central position underneath the flat nucleus. This positioning mechanism requires microtubule polymerization, as well as actin polymerization. We identify perinuclear actin structures that form in late G2/early prophase and interact with microtubules emanating from the centrosomes. Disrupting these structures by breaking the interactions of the linker of nucleoskeleton and cytoskeleton (LINC) complex with perinuclear actin filaments abrogates this centrosome positioning mechanism and causes an increase in subsequent chromosome segregation errors. Our results demonstrate how geometrical cues from the cell nucleus coordinate the orientation of the emanating spindle poles before nuclear envelope breakdown.

## Highlights

• Dynein and the actin/MT network coordinate centrosome positioning and separation
• The MT/actin network antagonizes Eg5-dependent separation
• LINC-complex-dependent perinuclear actin is critical for this mechanism
• Disrupting the LINC/actin interaction results in centrosome mis-positioning

## Graphical Abstract

**Figure:**
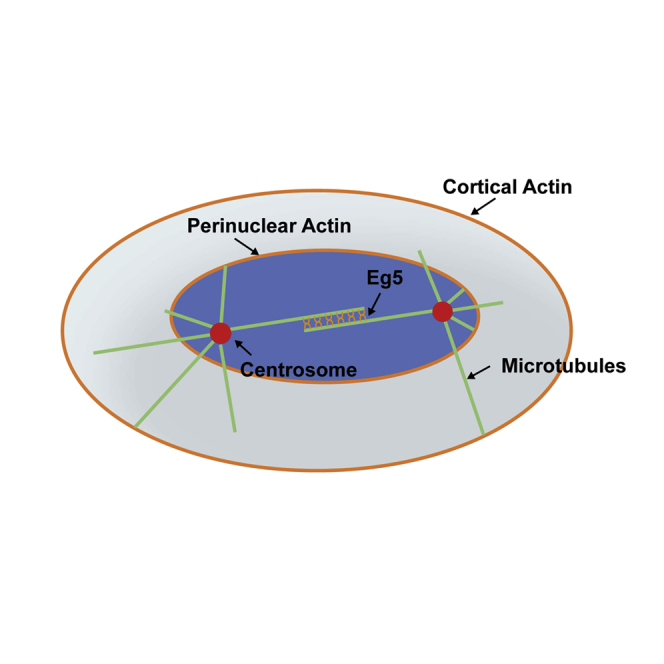

## Teaser

Stiff et al. describe how prophase-specific perinuclear actin in connection with polymerizing microtubules regulates the positioning of centrosome separation before nuclear envelope breakdown. This depends on the interaction of the LINC complex with actin. Breaking this link results in centrosome mispositioning and an increase in sister chromatid segregation errors.

## Introduction

Centrosomes are the microtubule (MT) organizing centers in animal cells. Centrosome numbers are restricted to one in G1/S and two in G2/M phase but are often amplified in cancer cells (Godinho, 2014). Following duplication, the two centrosomes stay closely linked by protein bridges involving C-NAP1 and Rootletin throughout S phase and G2 phase (Bahe et al., 2005, Mayor et al., 2000) but separate rapidly at the onset of mitosis to form the poles of the mitotic spindle (Conduit et al., 2015, Fu et al., 2015). To achieve separation, the centrosomes need to be actively pushed apart by the MT plus-end-directed kinesin Eg5 that generates force by cross-linking and sliding antiparallel MTs (Rosenblatt, 2005, Tanenbaum and Medema, 2010). Centrosome separation is not necessarily coordinated with nuclear envelope breakdown (NEBD) and can occur both in prophase and prometaphase (Kapoor et al., 2000, Kaseda et al., 2012, Rosenblatt et al., 2004, Silkworth et al., 2012, Whitehead and Rattner, 1998). During late G2/early prophase, centrosomes slide along the nuclear envelope (NE), while this association is lost after NEBD in pro-metaphase. Sufficient separation during prophase is not essential for bipolar spindle formation but is important for the establishment of accurate sister chromatid alignment. Both premature and insufficient separation of centrosomes before NEBD can cause an increase in sister chromatid attachment and segregation errors that may contribute to aneuploidy and tumorigenesis (Kaseda et al., 2012, Nam et al., 2015, Nam and van Deursen, 2014, Silkworth et al., 2012). Thus, understanding the balance of forces impacting on prophase centrosome separation is a critical question for our understanding of genome stability (Agircan et al., 2014).

Ideally, separated centrosomes should reach a symmetrical position along the diameter of the nuclear disc before NEBD to allow optimal amphitelic capture of the sister chromatids and bi-orientation of the kinetochores in prometaphase (Kaseda et al., 2012, Silkworth et al., 2012). The mechanisms that underlie the spatial coordination of centrosome separation are only poorly understood. Eg5-driven centrosome movement alone is not sufficient to explain how the separation process is spatially controlled. Dynein-dependent tethering of the centrosome to the NE via astral MTs is an important additional factor for positioning of the separating centrosomes at the NE (Gönczy et al., 1999, Robinson et al., 1999, Splinter et al., 2010). However, NE tethering, while important to keep the centrosome close to the chromosomes, may not be sufficient to explain potential spatial coordination of centrosome separation with regards to nuclear and cellular symmetry. During interphase centrosomes are subjected to forces that maintain a steady state positioning effect at the cellular centroid (Burakov et al., 2003, Théry et al., 2006) and these mechanisms are likely to contribute to spatial coordination of centrosome separation. However, a clear link between centrosome separation and the interphase positioning mechanism has not been established.

Two key factors have been proposed to account for maintaining the position of the centrosomes near the cell center. First, cortical Dynein pulls on the centrosome generating an overall centering force (Burakov et al., 2003, Zhu et al., 2010). Second, pressure built up by MT polymerization against the cortical cell periphery could also be a major contributor to centrosome positioning. This mechanism has been demonstrated *in vitro* (Faivre-Moskalenko and Dogterom, 2002, Holy et al., 1997, Pinot et al., 2009), *in silico* (Letort et al., 2016), and in fungi (Brito et al., 2005, Tran et al., 2001). Moreover, MCAK(mitotic centromere-associated kinesin, Kif2C/kinesin-13)- and Kif18B-(kinesin-8)-dependent MT depolymerization contributes to bipolar spindle formation when Eg5 is inhibited, suggesting an antagonism between spindle pole separation and MT polymerization in mitosis (van Heesbeen et al., 2017). The involvement of Tiam-1/Rac and p21-activated kinase signaling in opposing Eg5 could imply cross-talk with the cell cortex in this mechanism (Whalley et al., 2015, Woodcock et al., 2010). Accordingly, we have previously reported that inhibiting Cdk1 in chicken DT40 cells results in slow centrosome separation that is constrained by cortical MT pressure (Smith et al., 2011). DT40 cells are spherical lymphocytes with a limited cytoplasmic area surrounding the nucleus. Thus, the significant Eg5-antagonizing forces that we observed could be related to the special geometry of these cells. Overall, it remains to be determined if and to what extent MT polymerization and actin filaments impact pre-NEBD centrosome separation and if this mechanism contributes to spatial coordination of this process.

In this study, we characterized the coordination of centrosome separation and positioning before NEBD in human cells. We find that centrosome separation before NEBD is antagonized by forces that push the centrosomes toward a central position underneath the nucleus. The bulk of this mechanical force that counteracts centrosome separation requires MT and actin polymerization and acts differentially on the two centrosomes. In the absence of this antagonizing force, centrosome separation continues beyond the border of the NE and loses its symmetrical position relative to the nucleus. We observe the formation of transient G2/Prophase specific perinuclear actin structures that provide the geometrical coordination for this centering mechanism toward the nuclear centroid. Accordingly, disrupting the association of the linker of nucleoskeleton and cytoskeleton (LINC) complex with F-actin results in mispositioned centrosomes at NEBD correlating with increases in sister chromatid segregation errors.

## Results

### Centrosome Position before NEBD Is Stabilized by Eg5-Antagonizing Forces

**Figure 1:**
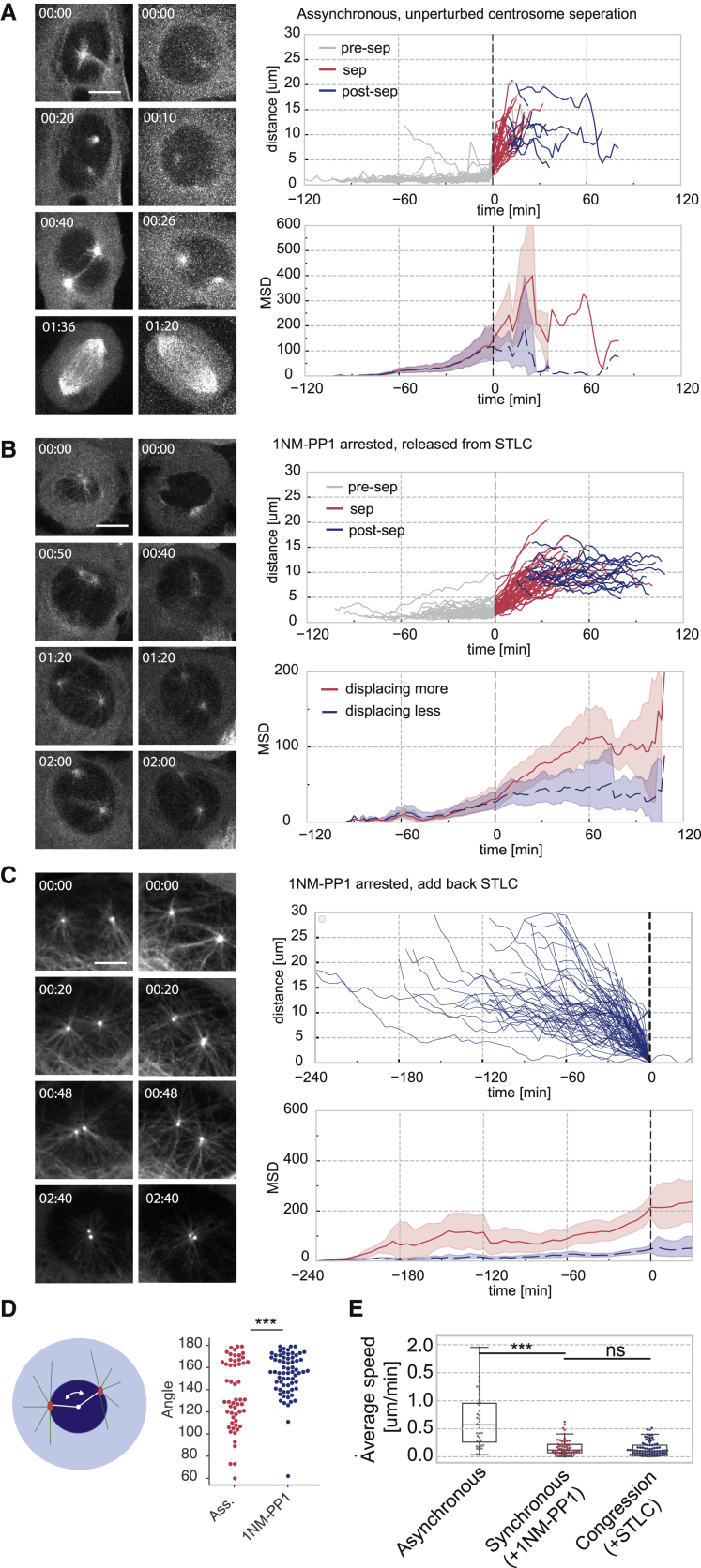
Centrosomes Reach a Stable Position along the Nuclear Diameter at NEBD (A) Centrosome separation in asynchronously dividing U2OS cdk1as cells (see also Video S1). Left panels show still images of two representative examples of centrosome separation in asynchronous cells. The cells stably expressed alpha-tubulin-GFP shown in white and RFP-PACT (not shown). Time is indicated in hours:minutes (h:min). The scale bar represents 10 μm and applies to each image. The top right panel shows centrosome distance (μm) over time (min) of single-cell experiments. The tracks were aligned by the time of separation. Tracks before separation are shown in gray, actively separating tracks are shown in red, and post-separation tracks are shown in blue. The bottom right panel shows the average mean square displacement (MSD) of each centrosome. For each cell, centrosomes were grouped into high displacement (red) and low displacement (blue), and the MSD for each group is plotted. The shaded area indicates the standard deviation. Tracking data are from three experiments with a combined n = 37. (B) Centrosome separation in 1NM-PP1-arrested U2OS cdk1as cells (see also Video S2). As in (A), but this time, cells were arrested for 20 h in 2 μM 1NM-PP1 and 5 μM STLC. The cells were washed 10 times in 1NM-PP1 medium without STLC before starting the imaging sequence. Tracking data are from three experiments with a combined n = 52. (C) Centrosome congression in 1NM-PP1-arrested U2OS cdk1as cells (see also Video S3). As in (A) and (B), but this time, cells were treated for 20 h in 1 μM 1NM-PP1 to allow centrosome separation to proceed. 5 μM STLC was added before initiating the imaging experiment. The still images in (A), (B), and (C) correspond to Videos S1, S2, and S3, respectively. Tracking data are from three experiments with a combined n = 80. (D) Quantification of centrosome angle with regard to the nuclear centroid. The diagram on the left indicates the procedure of this measurement. Nuclei and centrosomes were segmented, then nuclear centroid and centrosome angle were calculated based on the segmented data. The angles of centrosomes at NEBD in asynchronous cells and 2 h after STLC washout in 1NM-PP1-arrested cells are shown in the swarm plot (n = 120 per experiment). (E) Centrosome speed based on tracks shown in (A), (B), and (C). Speed was calculated as distance over time. For (D) and (E), p values were calculated using an independent two-sample t test. Levels of significance are indicated by stars (^∗^p < 0.05, ^∗∗^p < 0.01, **^∗∗∗^**p < 0.001). The boxplot indicates median, first and third quartiles, and minimum/maximum values.

To analyze the dynamics of centrosome separation prior to NEBD, we used U2OS cells that carry a Cdk1-analog-sensitive mutation (cdk1as). These cells arrest in late G2 phase with separated centrosomes before NEBD following Cdk1 inhibition by the bulky ATP analog 1NM-PP1 (Hochegger et al., 2007, Rata et al., 2018). Under both asynchronous and 1NM-PP1-arrested conditions, centrosomes mostly resided underneath the disk-shaped nuclear surface in the “fried egg” geometry of U2OS cells, reducing the geometry of the system to two dimensions. During unperturbed separation before NEBD, centrosomes often reached a position along the diameter of the nuclear disk with a distance of ∼10 μm (Figure 1A; Video S1). This was then followed by NEBD and spindle formation. We also measured the absolute centrosome movement by calculating the mean square displacement (MSD) of each centrosome (Figure 1A, bottom right panel). This analysis revealed a difference between the two centrosomes in both centrosome separation and congression, with one centrosome clearly displacing over a larger distance than the other. Unlike previously reported for asymmetric centrosome movement in early S phase (Piel et al., 2000), the slow and fast movement did not appear to correlate significantly with centrosome age (Figures S1A–S1C).

Video S1. Related to Figure 1A Centrosome separation in two representative asynchronous U2OS cdk1as cells stably expressing GFP-alpha-tubulin (shown in grgray). Time is indicated as hh:min, the scale bar represents 10μm.

We next analyzed centrosome separation in 1NM-PP1-treated cells that arrest in late G2-phase due to Cdk1 inhibition (Figure 1B; Video S2). For this purpose we suppressed centrosome separation with the Eg5 inhibitor S-trityl-L-cysteine (STLC) (Skoufias et al., 2006) and subsequently triggered centrosome separation by washing out STLC, while maintaining 1NM-PP1. We speculated that if pre-NEBD centrosome separation is positionally coordinated, centrosomes should reach a stable position along the diameter underneath the nuclear disk following separation in G2-arrested cells. Removal of STLC led to activation of Eg5 and resulted in a brief 30-min time window of constant centrosome movement toward a stable position close to the endpoint of the nuclear diameter with an average distance of ∼10 μm, similar to the position of centrosomes at NEBD in asynchronous cells. The centrosomes continued to undergo minor erratic movement (Video S2) but maintained this endpoint position without significant changes in distance and orientation. Similar to the measurements in asynchronous cells, centrosome movement in the 1NM-PP1-treated cells proceeded asymmetrically, with only one centrosome showing increased MSD (Figure 1B bottom left panel), but this asymmetry did not appear to correlate with centrosome age (Figure S1).

Video S2. Related to Figure 1B Centrosome separation in two representative U2OS cdk1as cells that were treated for 16 hours with 2μM 1NM-PP1 and 5μM STLC. Before imaging STLC was washed out in 1NM-PP1 containing medium. GFP-alpha-Tubulin is shown in white, time is indicated as hh:min, the scale bar represents 10μm.

We measured the precise alignment of the centrosomes at NEBD by calculating the angle between the centrosomes with regard to the nuclear centroid (Figure 1D). In the case of asynchronously dividing cells, this was above 90 degrees, with two clusters around 120 and 170 degrees. In the case of 1NM-PP1-arrested cells, most centrosomes reached an alignment closer to 180 degrees, indicating that they maintained a stable position close to the nuclear diameter. We hypothesized that this stable yet dynamic position could be maintained by Eg5-counteracting forces that act in a spring-like fashion and limit the Eg5-driven movement. To test this idea, we inhibited Eg5 by addition of STLC after separation occurred in the 1NM-PP1-arrested cells (Figure 1C; Video S3). This led to a quick collapse of the stable separated position and a reverse movement of the centrosomes toward each other, suggesting that a balance of Eg5-dependent and Eg5-antagonizing forces maintain the steady-state position prior to NEBD. This reverse movement, which we will refer to as centrosome congression, appeared to be the exact mirror image of separation in 1NM-PP1-arrested conditions, proceeding asymmetrically (Figure 1C, bottom left panel) and with similar speed (Figure 1E). The speed of centrosome separation in asynchronous cells was approximately twice as fast as the separation and congression speed in 1NM-PP1-arrested cells (Figure 1E), suggesting a significant additional impact for Cdk1 on separation dynamics. Overall, our data suggest that Eg5-antagonizing forces act on the separating centrosomes prior to NEBD.

Video S3. Related to Figure 1C Centrosome congression in two representative U2OS cdk1as cells that were treated for 16 hours with 2μM 1NM-PP. Before imaging 5μM STLC was added to the 1NM-PP1 containing medium. GFP-alpha-Tubulin is shown in white, time is indicated as hh:min, the scale bar represents 10μm.

### The Dynein/Kinesin-1 Balance Is Critical for Congression along the NE

**Figure 2:**
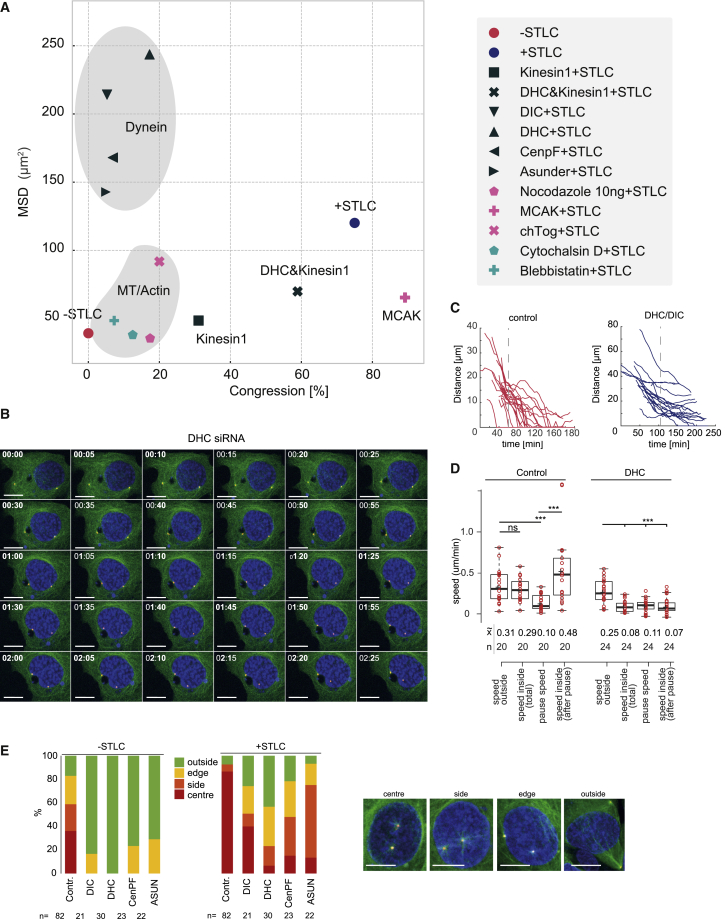
The Dynein/Kinesin-1 Balance Is Critical for Congression along the NE (A) Summary of MSD and congression data. Mean values of MSD (y axis) and percent congression (x axis) are plotted of all congression experiments performed in this paper. Dynein and MT/actin clusters are highlighted by gray areas. Individual data points are referenced in the figure legend to the right. See Figure S1 for confirmation of siRNA depletions. (B–D) Centrosome congression in DHC-depleted cells in cytoplasm and at the NE. (B) Images from time-lapse video of centrosome congression in DHC-depleted cells (green, GFP-α-tubulin; red, RFP-PACT; blue, Hoechst-33342-labeled DNA). Time is indicated in h:min on the top left, and scale bar represents 10 μm (see also Video S3). (C) Individual tracks of distance over time in control and DHC-depleted cells. Tracks are aligned along the time axes by the point of contact of both centrosomes with the nucleus. (D) Quantification of speed in the cytoplasm (outside) and nucleus (inside) 0–20 min after reaching the nucleus (pause) and t > 20 min after reaching the nucleus (after pause). See also Figure S2 for more data on dynein depletion and centrosome congression. p values were calculated using an independent two-sample t test. Levels of significance are indicated by stars (^∗^p < 0.05, ^∗∗^p < 0.01, **^∗∗∗^**p < 0.001). The boxplot indicates median, first and third quartiles, and minimum/maximum values. (E) Qualitative analysis of centrosome position before and 2 h after STLC treatment following indicated siRNA depletions. Centrosome position was scored as indicated in color legend (n indicates the number of live-cell imaging sequences analyzed). Examples of positions are shown in images below (scale bar, 10 μm).

Our analysis of pre-NEBD centrosome separation suggests the presence of a mechanism that antagonizes Eg5 and coordinates centrosome position with respect to the nucleus prior NEBD. Splinter et al. (2010) demonstrated that dynein/bicaudal-D2-dependent linkage of centrosomes to the NE maintains the association of centrosomes and the nucleus during G2 phase. However, one can expect additional mechanisms involving the cell cortex to coordinate the separating centrosomes with regard to cellular geometry. Thus, we focused our analysis on the action of dynein/kinesin-1 and the actin/MT network. We depleted or inhibited different components of these cytoskeletal systems and analyzed the effect of these perturbations on centrosome congression in 1NM-PP1-arrested U2OS cells. In Figure 2A, we summarize these data by plotting MSD against percentage of congression (for confirmation of depletion of individual proteins, see Figures S2C and S3B). The reference points of this analysis are congression assays with and without added STLC. Interfering with the dynein motor (depletion of dynein-heavy-chain [DHC] and dynein-intermediate-chain [DIC]) or dynein-NE association (depletion of CENPF and asunder) increases MSD but reduces congression, suggesting that movement of centrosomes has lost its overall direction but is still subjected to force. The contribution of dynein to centrosome congression could be derived from the cortex- and/or NE-associated pool of dynein. To distinguish between these two pools, we also analyzed centrosome congression in cells following asunder and EN CENPF depletion. These proteins are required for dynein nuclear association, but not for cortical association and motor function (Bolhy et al., 2011, Jodoin et al., 2012, Smoyer and Jaspersen, 2014). CENPF and asunder depletion caused a reduction in congression and an increase in MSD comparable to DHC or DIC depletion (Figure 2A).

Centrosome movement in dynein-depleted cells was unperturbed in the cytoplasm but appeared to stall upon reaching the vicinity of the NE (Figures 2B and S2A). We quantified this differential speed by aligning centrosome congression tracks from control and DHC-depleted cells by the time point when the centrosomes reached the NE (Figures 2C and 2D) These tracks reveal that centrosomes steadily travel through the cytoplasm, pause briefly when reaching the NE, and then continue their movement toward the nuclear centroid position. Upon DHC depletion, the centrosomes travel with comparable speed to controls in the cytoplasm but do not recover from stalling once reaching the NE. This phenotype can be also observed following depletion of CENPF or asunder (Figure 2E). In all cases, the centrosomes are dislocated from the NE in 1NM-PP1-arrested U2OS cdk1as cells and move toward the nucleus upon STLC treatment. However, unlike control cells, they do not continue congression to meet at the nuclear centroid position. This analysis suggests that dynein is not required for centrosome congression in the cytoplasm, but it seems to be critical to overcome a barrier to move toward the nuclear centroid underneath the NE.

Similar to previous reports (Splinter et al., 2010), we observe that the effects of dynein depletion on congression are negated by co-depletion of kinesin-1 (Figures 2A and S2B). Taken together, these data suggest that the main contribution of dynein to centrosome congression comes from the NE and that it acts predominantly by antagonizing kinesin-1.

### Centrosome Congression Depends on MT Polymerization

**Figure 3:**
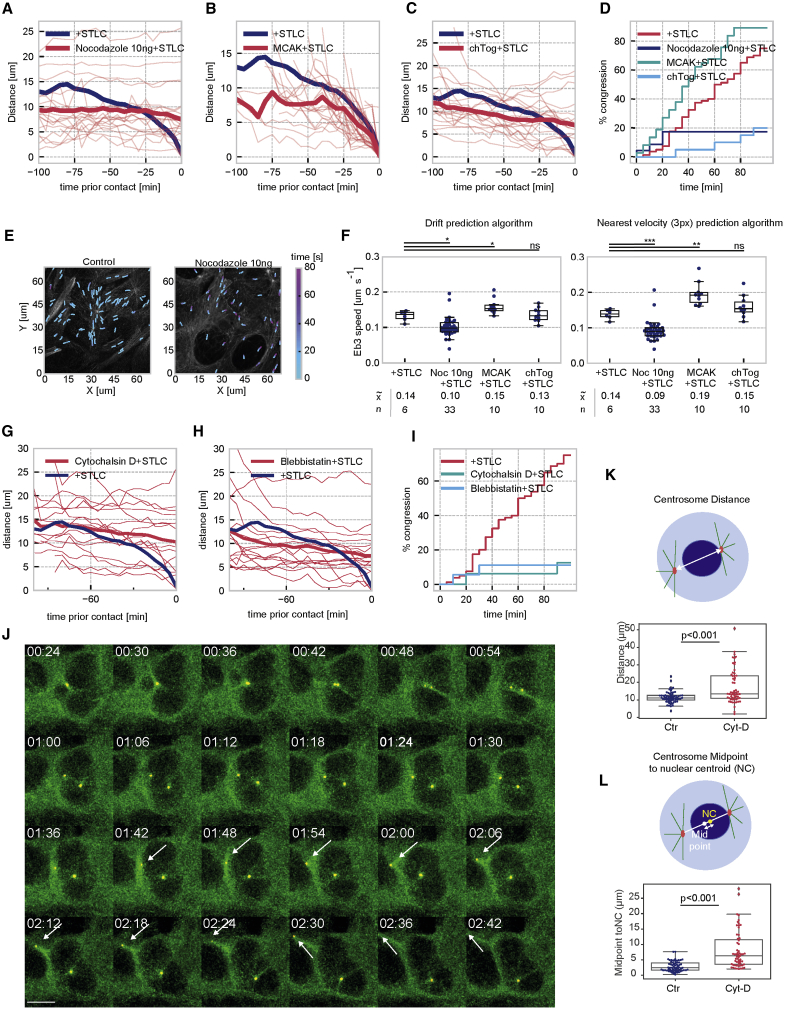
Centrosome Congression Depends on MT Polymerization (A–C) Measuring congression after manipulating MT polymerization Following 20-h arrest in 2 μM 1NM-PP1, cells with two separated centrosomes were followed by live-cell imaging after treatment with 5 μM STLC, and centrosome position was tracked to measure inter-centrosomal distance over time. (A) Cells treated with 10 ng/mL nocodazole. (B) Cells following 48-h MCAK siRNA depletion. (C) Cells following 48-h ch-TOG siRNA depletion. For all graphs, the bold blue line indicates the average congression tracks of control cells (see Figure 1). Individual tracks of cells are shown in red, and bold red lines represent the mean values. (D) Quantification centrosome congression. Data from (A)–(C) were used to calculate percentage of cells in which centrosomes joined together over time following the different treatments indicated in the legend. Each treatment was repeated in at least three independent experiments with total n = 23 (Noc), n = 20 ch-TOG), and n = 37 (MCAK). (E) EB3 comet tracking. Examples of MT tracking in RFP-EB3-expressing U2OS cdk1as cells following 20-h treatment with 1 μM 1NM-PP1. (F) Quantification of MT tracking. EB3 tracks in cells subjected to indicated treatments were analyzed using drift prediction and nearest speed tracking algorithms with a search radius of 3 pixels. Data were plotted for each condition as swarm/boxplots showing the mean of individual tracks per cell and the median plus 25 and 75 percentiles. p values were estimated using a two-sided t test. (G–I) Centrosome congression following changes in actin dynamics. As in Figure 2, U2OS cdk1as cells were treated for 20 h with 2μM 1NM-PP1, and cells with two separated centrosomes were analyzed by live-cell imaging and centrosome tracking following STLC treatment to measure inter-centrosomal distance over time. Cells were pretreated for 1 h with (G) 2 μg/mL cytochalasin D and (H) 5 μM blebbistatin. For all graphs, the bold blue line indicates the average congression tracks of control cells (see Figure 1). Individual tracks of cells are shown in red, and bold red lines represent the mean values. (I) Quantification centrosome congression. Data from (A)–(C) were used to calculate percentage of cells in which centrosomes joined together over time following the different treatments indicated in the legend. Each treatment was repeated in at least three independent experiments with total n = 18 (Blebbistatin), n = 16 (Cytochalasin-D). (J) Centrosome separation in cytochalasin-D-treated cells (see also Video S4). Images of time-lapse video showing centrosome separation (green, GFP-alpha-tubulin; red, RFP-PACT; time is indicated in h:min on the top left, and scale bar represents 10 μm) following STLC washout in cells that were treated with 2 μM 1NM-PP1 and 5 μM STLC for 20 h. Following an hour-long pretreatment with cytochalasin D, cells were washed with STLC-free medium before imaging. (K) Quantification of centrosome distance. Maximal distance was measured 2 h after STLC release. (L) Quantification of centrosome alignment. Geometrical alignment of centrosomes toward the nuclear centroid was measured as the distance from the midpoint between centrosomes to the nuclear centroid position (see diagram for graphic representation). Data in (F) and (G) are from three experiments (total n > 50 for each condition). For (F), (K), and (L), p values were calculated using an independent two-sample t test. Levels of significance are indicated by stars (^∗^p < 0.05, ^∗∗^p < 0.01, **^∗∗∗^**p < 0.001). The boxplot indicates median, first and third quartiles, and minimum/maximum values.

Figure 2A shows that the most significant effects on the congression movement of centrosomes were observed after perturbing MT and actin dynamics. We attempted to reduce MT polymerization with low-dose (10 ng/mL) nocodazole treatment and depletion of the MT polymerase CKAP5/- ch-TOG (Charrasse et al., 1998, Gergely et al., 2003) and enhanced polymerization by depletion of the MT depolymerase MCAK (Howard and Hyman, 2007, Wordeman and Mitchison, 1995). Nocodazole treatment (Figure 3A) and chTog depletion (Figure 3B) both severely inhibited centrosome congression, while centrosome congression proceeded faster in MCAK-depleted cells (Figure 3C). This was also reflected by the increased (MCAK) and reduced (ch-TOG/Nocodazole) percentage of cells with fully congressed centrosomes (Figure 3D). To correlate these results more directly with MT polymerization rates, we established U2OS cdk1as cells that stably expressed mCherry-EB3 (Figure 3E) and measured EB3 movement using two separate tracking algorithms (see STAR Methods). The low-dose nocodazole treatment caused a significant reduction in the length of EB3 tracks, while MCAK treatment caused an increase in the speed of EB3 comet movements, suggestive of increased polymerization rates (Figure 3F). Depletion of ch-TOG, on the other hand, did not appear to affect MT polymerization rates (Figure 3F), as was previously reported for its binding partner, Tacc3 (Gutiérrez-Caballero et al., 2015). This protein is thus likely to play a distinct role in coordinating MT dynamics and contributes to Eg5-antagonizing forces independently of MT growth rates. We also compared MT dynamics in G2-arrested and asynchronous cells with separated centrosomes to investigate if the 1NM-PP1 arrest impacts levels of MT polymerization. However, this did not appear to be the case, and MT polymerization rates in the two conditions occurred at a similar rate (Figure S3A).

### Actin Restrains Centrosome Separation in G2-Arrested Cells

Similar to MT dynamics, we also observed a significant reduction in centrosome displacement and congression (Figure 2A) following inhibition of actin plus-end polymerization by cytochalasin D (Cooper, 1987) and Myosin inhibition by blebbistatin (Straight et al., 2003). These treatments abolished centrosome congression to an extent comparable to nocodazole-treated cells, suggesting that actin plays a critical role in restraining Eg5-dependent separation (Figures 3G–3I). This observation allowed us to test the impact of Eg5-antagonizing force on centrosome separation and positioning. If this mechanism contributes to the positioning of centrosomes at NEBD, one can expect changes in the steady-state position of separated centrosomes. We tested this by performing centrosome separation assays in 1NM-PP1-treated U2OS cdk1as cells in the presence or absence of cytochalasin D (Figures 3J–3L). Similar to the experiments shown in Figure 1B, centrosomes in the DMSO-treated controls quickly reached a symmetrical position near the NE along the diameter of the nuclear disk. In cytochalasin-D-treated cells, this symmetry was lost, and centrosomes moved toward the side of the nucleus, with one of them often losing connection to the NE and continuing to migrate over large distances (Figure 3J; Video S4). Quantification of these results shows that both distance (Figure 3K) and symmetric alignment along the diameter of the nuclear disc (Figure 3L) are disrupted in a significant proportion of separated centrosomes following cytochalasin D treatment. Taken together, these results suggest that actin is critical to restrain Eg5-driven centrosome separation and coordinate the positioning of the separating centrosomes.

Video S4. Related to Figure 3E Centrosome separation following STLC washout in STLC/1NM-PP1 arrested U2OS cdk1as cells (as in Video S2). GFP-alpha-Tubulin is shown in green and RFP-PACT in red. The scale bar indicates a length of 10μM, time is indicated as hh:min on the top left. Left panel STLC wash out in control cells, right panel STLC washout in Cytochalasin D treated cells.

### The Eg5-Antagonizing Forces Push Centrosomes toward a Central Position underneath the Nucleus

**Figure 4:**
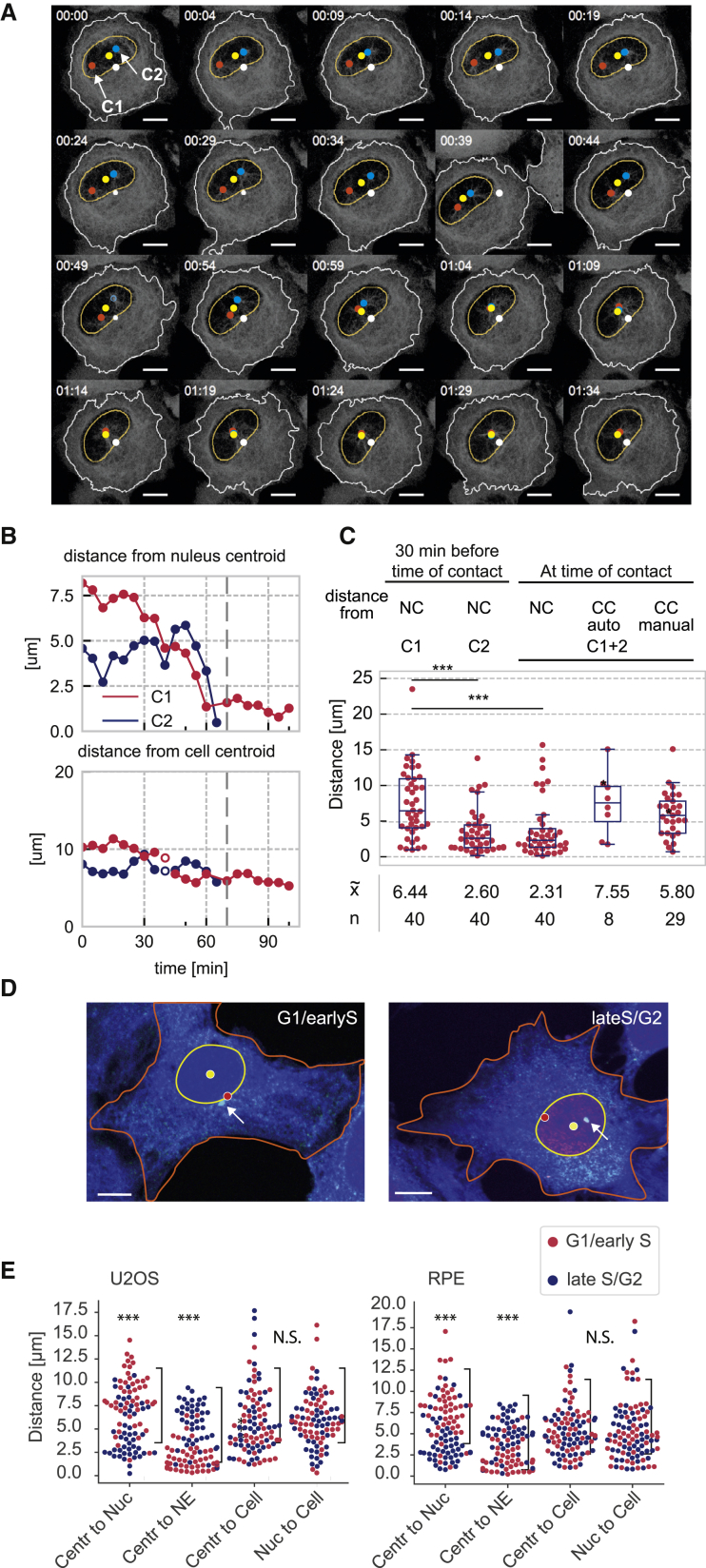
Centrosomes Congress toward the Nuclear Centroid (A) Automated tracking of centrosome position (see also Video S5). Still images describing the tracking of centrosome position using nuclear and cellular centroid as a reference. Nuclear and cellular masks were generated by automated image segmentation (see STAR Methods), the centroid was determined, and centrosome distance relative to nuclear and cellular centroid (yellow dot for nuclear and white dot for cellular centroid) was calculated using vector addition. Time is indicated in h:min on the top left; scale bar represents 10μm. (B) Quantification of tracking data. Centrosome tracks from the time-lapse experiment in (A) showing distance from nuclear and cellular centroid (μm) for centrosome 1 (C1, red) and centrosome 2 (C2, blue); dashed gray line indicates the time point when C1 and C2 make contact. (C) Data from population analysis. Distance from centroids 30 min before and at the time of contact was estimated as shown in (A) (nuclear centroid [NC], 32 tracks; cellular centroid [CC], 8 tracks). For cellular centroid estimation, automated segmentation was successful in only eight cells (due to problems with distinction from neighboring cells). Manual estimation of cellular centroid was performed at time of contact for 29 more tracks as indicated. p values were calculated using an independent two-sample t test. Levels of significance are indicated by stars (^∗^p < 0.05, ^∗∗^p < 0.01, **^∗∗∗^**p < 0.001). The boxplot indicates median, first and third quartiles, and minimum/maximum values. (D) Analysis of nuclear positioning in U2OS cells by immunofluorescence. G2 cells were identified by CENPF staining (red), cell shape was visualized using CellTracker blue, and centrosomes were detected using gamma-tubulin staining (green). Nuclear mask and centrosomes were segmented in Python using the skimage library, while cell shape was estimated manually. Images show centrosome position in G1/early S (CENPF-negative) and late S/G2 (CENPF-positive) U2OS cells. The nuclear centroid is shown in yellow, and the cellular centroid is shown in red; centrosomes were stained using gamma-tubulin antibodies (green) and are indicated by arrow. The scale bar indicates 10 μm. (E) Quantification of centrosome position in U2OS and RPE cells. U2OS and RPE cells were analyzed as described in (D) and plotted grouping CENPF-negative (G1/early S) cells in red and CENPF-positive (late S/G2) cells in blue (three experiments, n = 50 per group and experiment). The data show distances (in μm) for centrosome to nuclear centroid, centrosome to NE, centrosome to cellular centroid, and nuclear centroid to cellular centroid. Significance for differences between the CENPF-positive and negative groups for each measurement was calculated using a two-sided t test.

If the actin cortex is involved in spatial coordination of Eg5-dependent centrosome separation, it is likely to exert a centering force that pushes the centrosomes back toward the cell center. To test this hypothesis, we quantified the precise position of the centrosomes in 1NM-PP1-arrested cells as they congress following Eg5 inhibition (Figures 4A–4C; Video S5). We segmented nucleus and cell shape and calculated cellular and nuclear centroid position as a reference point relative to each centrosome (Figure 4A). Using this approach, we found that the point of contact in the majority of cells was nearer the nuclear centroid than the cell center (Figures 4B and 4C). To further analyze the relation of centrosome and nuclear position in asynchronous cells, we compared the centrosome position in G1/early S and late S/G2 phase in fixed U2OS and RPE cells by immunofluorescence (Figures 4D and 4E). We used CENPF staining as a marker for late S/G2 cells (Landberg et al., 1996) and manually measured the distance of centrosomes from cell centroid, nuclear centroid and NE, as well as the distance of cell and nuclear centroid in CENPF-negative (G1/early S) and positive (late S/G2) cells (Figure 4E). These results show that there is indeed a preference for G2 centrosomes to reside close to the nuclear centroid position, while the edge of the nucleus is preferred in G1 and early S phase cells. The distance between nuclear and cellular centroid did not change according to this analysis, suggesting that the positional change is based on movement of the centrosomes and not the nucleus. These results are very similar in U2OS and RPE-1 cells, suggesting that the positioning of centrosomes near the nuclear centroid in G2 phase is not a cell-type-specific phenomenon.

Video S5. Related to Figure 4A Centrosome congression toward the nuclear centroid. Centrosomes (red and blue) were tracked relative to nuclear mask (yellow) and cell mask (white) and distances from nuclear centroid (yellow dot) and cellular centroid (white dot) were calculated for each frame. Note the significant difference of movement between the two centrosomes and overlap of the meeting point with the nuclear centrosome.

### Prophase-Specific Perinuclear Actin Structures

**Figure 5:**
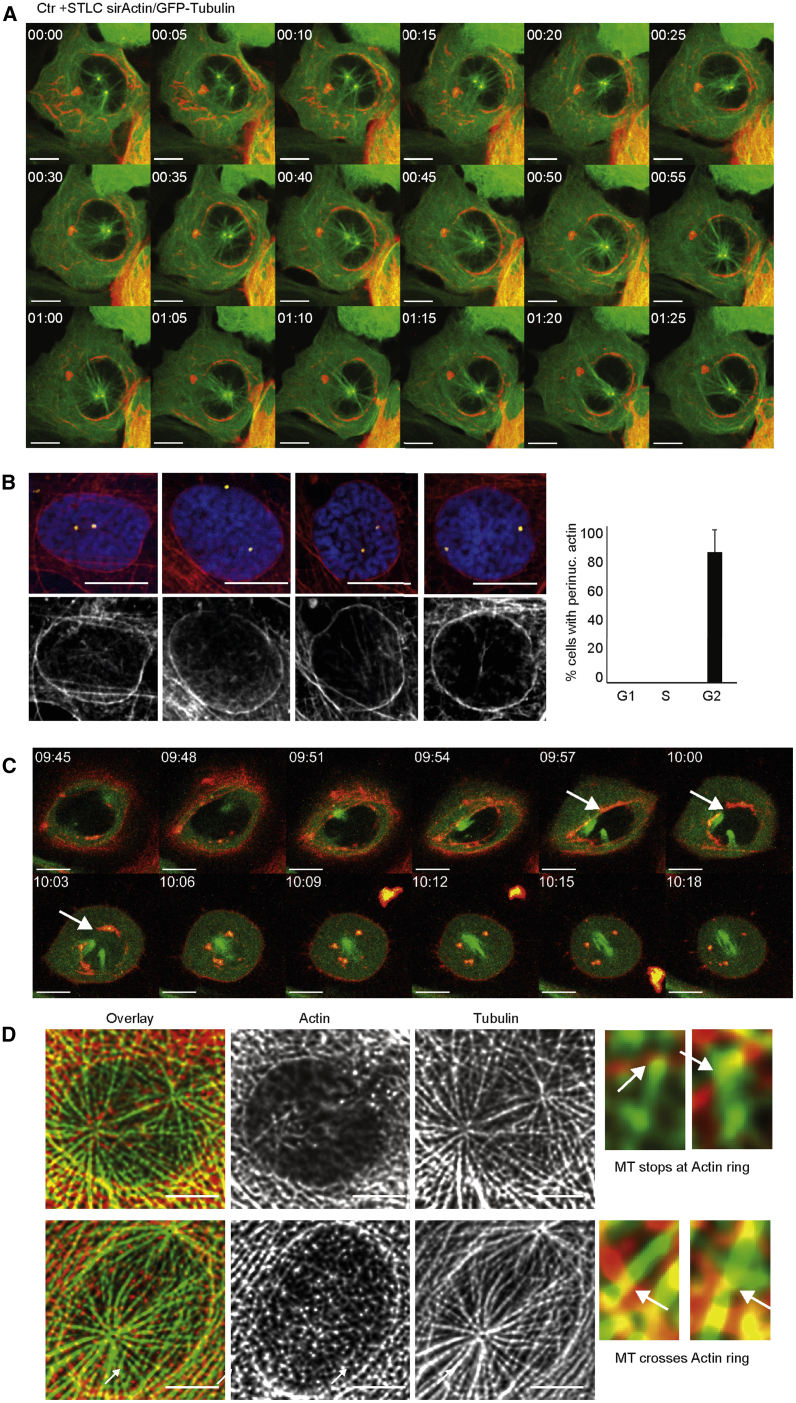
Prophase-Specific Perinuclear Actin Structures Coordinate Centrosome Positioning and Restrain Eg5-Dependent Separation (A) Perinuclear actin interacts with centrosomal MT asters (see also Video S6). Images from time-lapse video of centrosome congression following STLC treatment of 1NM-PP1-arrested U2OS cdk1as cells showing perinuclear actin structures (green, GFP-alpha-tubulin; red, SiR-actin; scale bar, 10 μm). (B) Phalloidin staining of G2/prophase-specific perinuclear actin in fixed U2OS cells. Images of fixed wild-type (WT) U2OS probed by immunofluorescence and phalloidin staining. The panels show examples of individual prophase cells. Top panels show overlays (phalloidin, red; gamma-tubulin, green; and DAPI, blue). The bottom panel shows the phalloidin staining (scale bar, 10 μm). The graph on the right shows the average percentage of cells with perinuclear actin structures in indicated cell-cycle phases (three experiments, n > 30, error bars indicate standard deviation). (C) G2/prophase-specific perinuclear actin in asynchronous U2OS cells detected by SiR-actin in living cells (see also Video S7). Images from a time-lapse movie in asynchronous U2OS cells showing formation of perinuclear actin structures in prophase (green, GFP-alpha-tubulin; red, SiR-actin; scale bar, 10 μm). See also Video S7. (D) Imaging of MT/actin interactions by SRRF processing. 1NM-PP1-arrested U2OS cdk1as cells were labeled by immunofluorescence and imaged using the SRRF technique. Phalloidin is shown in red and tubulin in green in the overlay. The smaller panels on the right show examples of MTs either stopping at or crossing the perinuclear actin ring. Scale bar, 5 μm.

Our results suggest that both MT polymerization and actin polymerization and contractility generate an Eg5-antagonizing force that pushes the centrosomes toward a position near the centroid underneath the nuclear disc and not toward the cellular centroid. The question of how actin-dependent forces transmit spatial information relating to nuclear symmetry arises. To address this question, we visualized both actin and MTs simultaneously during centrosome congression in 1NM-PP1-arrested U2OS cdk1as cells. We observed a prominent perinuclear actin ring that appeared to make contact with the MT asters that were emanating from the centrosomes (Figure 5A; Video S6). This structure could explain how MT/actin-dependent forces direct centrosome positioning toward the nuclear centroid.

Video S6. Related to Figure 5A Actin/MT interactions during centrosome congression in U2OS cdk1as cells that were treated for 16 hours with 2μM 1NM-PP1. 5μM STLC was added at the start of the imaging sequence (as in Video S3). GFP-alpha-Tubulin is shown in green and SiR-Actin in red. The scale bar indicates a length of 10μM, time is indicated as hh:min on the top left. Note the MT reaching toward the perinuclear actin ring.

Accumulation of perinuclear actin could be a consequence of the prolonged G2 arrest in the 1NM-PP1-arrested cells, or it could be caused by the SiR-actin probe (see STAR Methods). To address the physiological relevance of perinuclear actin, we analyzed prophase-specific perinuclear actin structures in asynchronous U2OS cells using phalloidin staining (Figure 5B). Prophase cells were scored based on the presence of separated centrosomes and condensed chromosomes within an intact nucleus. This allowed us to readily detect perinuclear actin structures surrounding the nucleus at this cell-cycle stage. Perinuclear actin was only detected in cells that were categorized as prophase and did not occur in other cell-cycle stages (Figures 5B and S4A). Moreover, blocking actin plus-end polymerization by cytochalasin D treatment and pretreatment of the cells with 20 ng/mL nocodazole abolished perinuclear actin (Figure S4A), while MCAK and DHC depletion by small interfering RNA (siRNA) did not prevent the formation of this structure (Figure S4B). We detected similar prophase-specific perinuclear actin structures in phalloidin-stained RPE-1 cells (Figure S4C), as well as in breast epithelial MCF10A cells and HCT116 colon cancer cells (Figure S4D). However, we failed to observe perinuclear actin in primary fibroblasts. This could be due to excessive stress fibers in these cells that mask the signal around the nucleus (Figure S4D). Another recent study reported similar prophase-specific perinuclear actin in U2OS and RPE-1 cells, but not in HeLa cells (Booth et al., 2019). Further work will be necessary to investigate what determines the absence or presence of this cytoskeletal arrangement across different cell lines. To better capture the transient nature of these structures, we labeled actin using the SiR-actin probe in asynchronous U2OS cells that were expressing tubulin-GFP. Based on this approach, we could detect an accumulation of peri-nuclear actin in cells that were about to enter mitosis. This structure was highly transient and rapidly dissipated following NEBD (Figure 5C and Video S7). To visualize the interactions of MTs emanating from the centrosome and the perinuclear actin ring, we analyzed 1NM-PP1-arrested cells using the Super Resolution Radial Fluctuations (SRRF) method (Gustafsson et al., 2016) (Figure 5D). This revealed a radial MT array that extended from the separated centrosomes toward the NE. We could readily observe MTs that appeared to connect to the actin ring, while others reached across this structure toward the cortex.

Video S7. Related to Figure 5C Prophase specific perinuclear actin formation in asynchronous U2OS cells, see also Figure 7G. GFP-alpha-Tubulin is shown in green and SiR-Actin in red. The scale bar indicates 10μm, time is indicated as hh:min on the top left. Note the transient formation of a perinuclear actin structure in prophase during time-frames 9:51 to 10:12

### Preventing LINC-Complex/Actin Interaction Disrupts Perinuclear Actin Formation and Correct Centrosome Positioning

**Figure 6:**
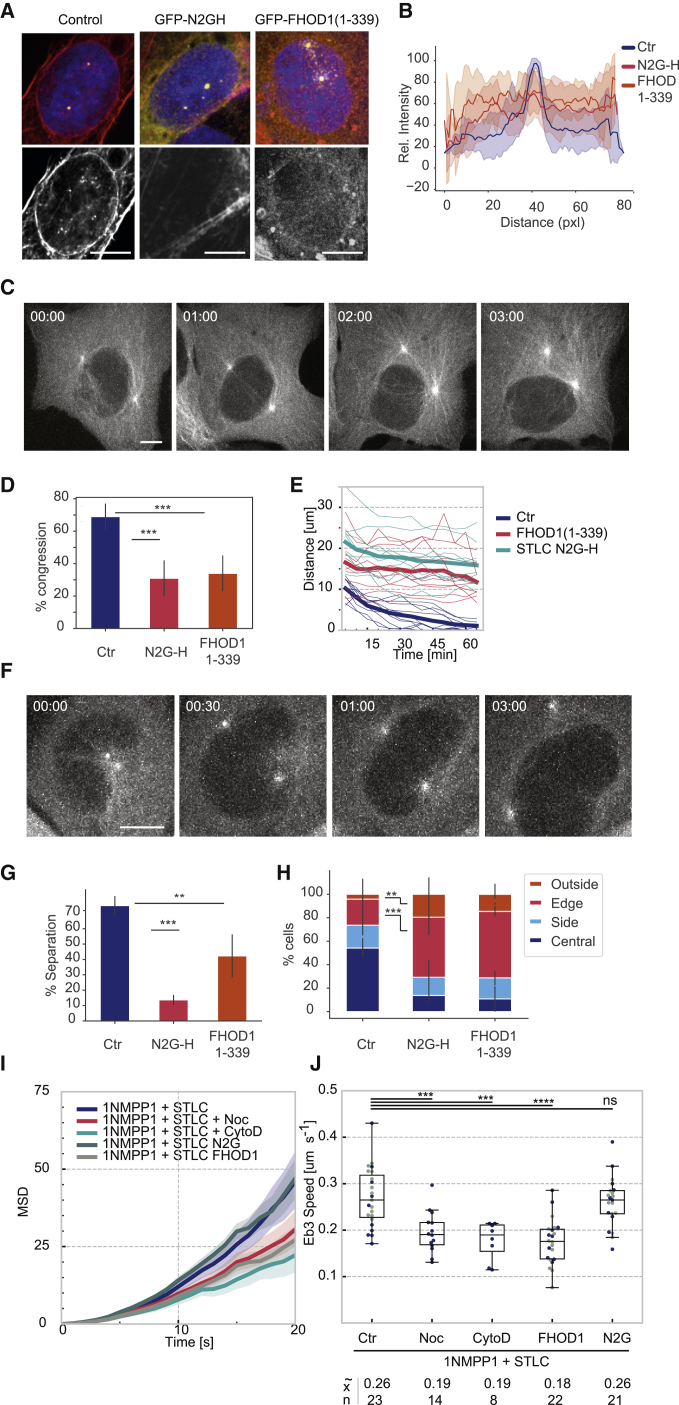
Disrupting LINC/Actin Interactions Disrupts Centrosome Separation and Positioning (A) Effects of GFP-N2G-H and GFP-FHOD1(1–339) expression on perinuclear actin. U2OS cdk1as cells were transiently transfected with GFP, GFP-N2G-H, and GFP-FHOD1(1–339) expression vectors. 24 h after transfection, the cells were arrested in G2 phase by treatment with 2 μM 1NM-PP1 for 20 h and then fixed and stained with phalloidin (red), pericentrin (green), and DAPI (blue). The scale bar indicates 10 μm. Top panels show overlays, and the bottom panel shows the phalloidin staining in black and white (b/w). (B) Quantification of perinuclear actin. Intensity profiles were measured in ImageJ along manually generated regions of interest (ROIs) representing a line crossing the NE. The highest intensity of each line was taken as 100%, and relative intensities are plotted. Bold lines indicate the mean values and the shaded areas the standard deviation (3 experiments, total n = 20 cells per condition). (C–E) Effects of GFP-N2G-H and FHOD1(1–339) on centrosome congression (see also Video S8, right panel). Centrosome congression assays were performed as described in Figure 1 in cells transiently expressing GFP, GFP-N2G-H, or FHOD1(1–339). (C) Representative example of a GFP-FHOD1(1–339) expressing cell. Images show SiR-tubulin-labeled MTs at indicated time points (h:min) following treatment with 5 μM STLC. (D) Quantification of percent of congression in GFP, GFP-N2G-H, or FHOD1(1–339) expression cells (mean of three experiments is shown, and the error bars indicate standard deviation; n > 50 per experiment). (E) Tracks of centrosome congression over 60 min following STLC treatment (10 individual tracks are shown, and the mean is indicated by thicker line; n > 50 per experiment). The scale bar indicates 10 μm. (F–H) Effects of GFP-N2G-H and FHOD1(1–339) on centrosome separation (see also Video S8, left panel). Centrosome separation was assayed in 1NM-PP1-arrested U2OS cdk1as cells transiently expressing GFP, GFP-N2G-H, or FHOD1(1–339) following release from STLC as described in Figure 1. (F) Representative example of a GFP-FHOD1(1–339)-expressing cell. Images show SiR-tubulin-labeled MTs at indicated time points (h:min) following release from STLC treatment. (G) Quantification of percent separation in cells expressing GFP, GFP-N2G-H, or FHOD1(1–339) (mean of three experiments is shown, the error bars indicate standard deviation, n > 50 per experiment). (H) Qualitative analysis of centrosome positioning 3 h following STLC release. In controls most centrosomes were aligned along the nuclear diameter underneath the nucleus or at the border of the NE. GFP-N2G-H and FHOD(1–339) expressing cells mostly failed to reach this position and resided toward the side of the nuclear border (mean of three experiments is shown, and error bars indicate standard deviation; n > 50 per experiment). The scale bar indicates 10 μm. (I and J) Effects of cytochalasin D, GFP-N2G-H, and FHOD1(1–339) on MT polymerization. RFP-EB3-expressing U2OS cdk1as cells were arrested for 20 h in 2 μM 1NM-PP1 and 5 μM STLC, treated with 2 μg/mL cytochalasin D and 20 ng/mL nocodazole, or transfected with GFP-N2G-H or FHOD-1 (1–339) expression vectors 24 h before arrest. 50 EB3 comets per cell were tracked manually, and comet speed and MSD were calculated. Median speed (x) and number of analyzed cells per condition (n) are indicated. For (D), (G), (H), and (J), p values were calculated using an independent two sample t test. Levels of significance are indicated by stars (∗p < 0.05, ∗∗p < 0.01, ∗∗∗p < 0.001). The boxplot indicates median, first and third quartile, and minimum/maximum values.

To analyze the role of perinuclear actin structures in nuclear centrosome positioning and separation, we aimed to specifically disrupt the NE-associated actin pool. The LINC complex (Razafsky et al., 2011, Starr and Fridolfsson, 2010) plays an essential role in the contact between actin and the NE, and this requires the interaction of the LINC component nesprin-2G with the diaphanous-related formin FHOD1 (Antoku et al., 2015, Kutscheidt et al., 2014). Expression of fragments that contain only the respective docking sites of N2G (N2G-H) or FHOD-1 (FHOD1(1–339)) results in the disruption of these complexes (Kutscheidt et al., 2014). We hypothesized that these dominant-negative domains could also disrupt the formation of G2-specific perinuclear actin fibers that we observed in 1NM-PP1-arrested U2OS cells. Indeed, transient expression of N2G-H and FHOD1(1–339) fused to GFP at the respective N termini resulted in an accumulation of these proteins at the border of the NE (Figure S5A) and a marked reduction of perinuclear actin filaments in 1NM-PP1-treated U2OS cdk1as cells (Figures 6A and 6B). Overexpression of these domains did not have an apparent effect on the cell shape and cortical actin (Figure S5B). However, the presence of both FHOD1(1–339) and N2G-H significantly reduced the congression of separated centrosomes following Eg5 inhibition (Figures 6C–6E; Video S8). Moreover, the centrosome position was clearly shifted away from the NE. The distance between separated centrosomes in 1NM-PP1-arrested U2OS cells increased twofold, and a majority of these cells failed to congress upon STLC treatment (see tracks in Figure 6E). These data suggest that the disruption of NE-actin interactions causes a reduction in the Eg5-antagonizing forces that push the centrosomes toward the nuclear centroid.

Video S8. Related to Figures 6C and 6F Effects of GFP-FHOD(1-339) expression on centrosome congression (left panel, as in Video S3) and separation (right panel, as in Video S2) in 1NM-PP1 arrested U2OS cdk1as cells. GFP-alpha-Tubulin is shown in white, the scale bar indicates 10μm, time is indicated as hh:min on the top right.

When centrosomes separated in 1NM-PP1-arrested cells expressing these proteins, the usual stable steady-state position along the nuclear diameter could not be maintained, and association with the NE was often lost (Figures 6F–6H; Video S8). However, we found that overexpression of the dominant-negative N2G-H and FHOD1(1–339) domains also caused a significant reduction in centrosome separation when cells were released Eg5 inhibition (Figure 6G). This suggests that NE-associated actin cables are involved not only in centrosome positioning but also in supporting Eg5-dependent separation.

Given the substantial cross-talk between MT and actin cytoskeleton, we analyzed the impact of manipulating global actin polymerization by cytochalasin D and perinuclear actin by N2G-H and FHOD1(1–339) expression on MT dynamics. For this purpose, we measured the speed and MSD of EB3 comets (Figures 6I and 6J). These data show that cytochalasin D treatment and FHOD1(1–339) expression reduce MT polymerization to a level comparable to low-dose nocodazole treatment. However, N2G-H expression did not affect EB3 comet speed and displacement. This suggests that disruption of the nesprin/actin interaction affects centrosome dynamics independently of MT polymerization rates, and it also highlights the extensive level of cross-dependence of MT and actin dynamics.

### Expression of Dominant-Negative FHOD1 and Nesprin N2G-H Disrupts Centrosome Positioning at NEBD and Causes Sister Chromatid Segregation Errors

**Figure 7:**
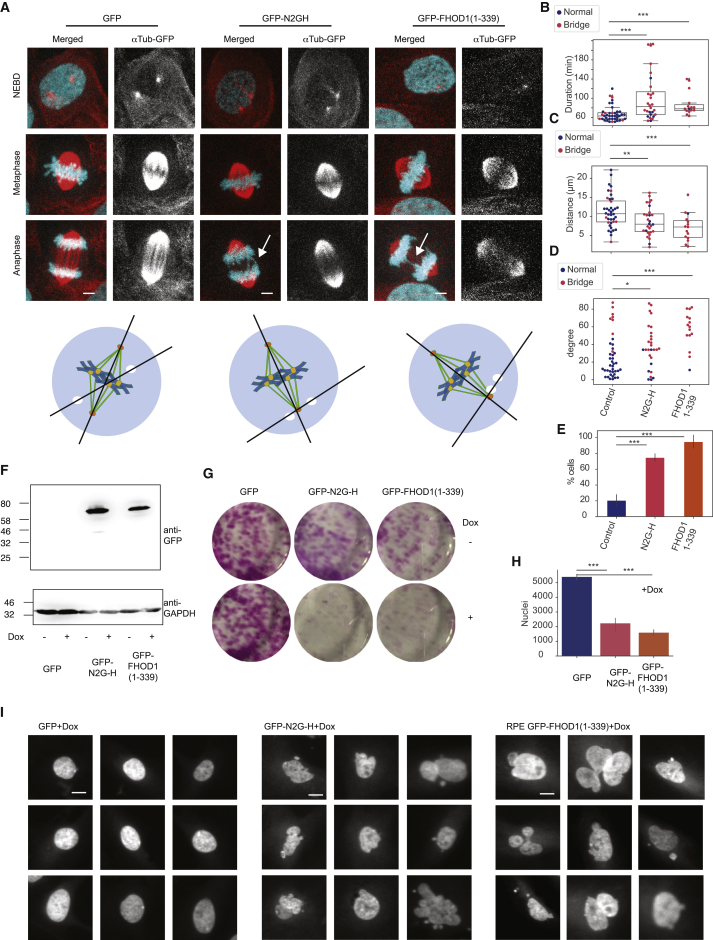
Expression of Dominant-Negative GFP-Nesprin N2G-H and FHOD1(1–339) Causes Centrosome Separation and Positioning Defects and Chromosome Segregation Errors in Asynchronously Dividing U2OS Cells (A) Effects of GFP-N2G-H and FHOD1(1–339) domains on centrosome position and sister chromatid segregation Representative images of cells transiently expressing GFP, GFP-N2G-H, and GFP-FHOD1(1–339). The time-lapse images show the same cell at NEBD, metaphase, and anaphase. The overlays show histone H2B-fusionRed in blue and SiR-tubulin in red. Black and white images show single-channel images of SiR-tubulin. The arrows indicate lagging chromosomes, and the scale bar represents 5 μm. The diagrams below the image panels show the measurement of the axis between centrosomes at NEBD and the spindle poles at metaphase. This was used to estimate the degree of spindle rotation between NEBD and metaphase in (D). (B–D) Quantitative analysis of mitotic phenotypes. Mitotic progression was analyzed from three transient transfection experiments (total n = 40 for GFP, 30 for GFP-N2G-H, and 20 for GFP-FHOD1). Each cell was classified according to the presence (red dots) or absence (blue dots) of chromosome bridges in anaphase. (B) Quantification of the time spent between mitotic entry and anaphase. (C) Distance between the centrosomes at NEBD. (D) Angle between the centrosome axis at NEBD and the spindle pole axis at metaphase as indicated in the diagrams in (A). p values for (B) and (C) were estimated using a two-sided t test. p values for (D) were estimated using a Mann-Whitney test due to the non-normal distribution of the angle measurements. The boxplot indicates median, first and third quartiles, and minimum/maximum values. (E) Quantification of percentage of cells that display anaphase chromosome bridges. (F–I) Inducible expression of GFP-N2G-H and FHOD1(1–339) domains in RPE-1 cells. (F) RPE-1 cells were engineered to stable express GFP-N2G-H and GFP-FHOD1(1–339) from a doxycycline-inducible promoter using the sleeping beauty transposon system. Induction of protein expression was confirmed 24 h after addition of 2 μg/mL doxycycline to the growth medium by immunoblotting with GFP antibodies. (G) Colony-formation assays of RPE-1 cells expressing the indicated cDNAs from the inducible promoter in growth medium with or without 2 μg/mL. (H) Quantification of cell proliferation following 5-day growth in 2 μg/mL doxycycline addition by nuclei counting. (I) Display of representative Hoechst-stained nuclei in RPE-1 cells expressing GFP, GFP-N2G-H, or GFP-FHOD1(1–339) 5 days after doxycycline induction (scale bar, 10 μm). For (E) and (H), p values were calculated using an independent two sample t test. Levels of significance are indicated by stars (∗p < 0.05, ∗∗p < 0.01, ∗∗∗p < 0.001). Error bars show standard deviation.

We analyzed the effect of GFP-N2G-H and GFP-FHOD1(1–339) expression on centrosome separation and positioning in asynchronously dividing cells (Figure 7). Overexpression of these dominant-negative domains resulted in a delay in mitotic progression and in a marked increase in anaphase chromosome bridges (Figures 7A and 7B). When analyzing centrosome separation at NEBD, we noticed two distinct phenotypes at this stage. First, the distance of separated centrosomes was markedly reduced (Figure 7C), in accordance with our previous observation on reduced centrosome separation following release from STLC (Figure 6G). Moreover, centrosomes were also mis-oriented at NEBD, resulting in significant changes in the orientation of the mitotic spindle following NEBD compared to the centrosome position at NEBD (see diagram in Figure 7A). We observed a notable correlation in this positioning effect with the occurrence of anaphase chromosome bridges. Thus, even in control cells, an increase in the angle between the centrosome position at NEBD and the spindle position at the metaphase anaphase transition appeared to correlate with the occurrence of anaphase chromosomes bridges (red dots in Figures 7B–7D and bar plots in Figure 7E). Expression of N2G-H and FHOD1(1–339) resulted in a much higher proportion of cells with an increase in this angle, and most of these cells also displayed chromosome bridges in anaphase (Figure 7D). Conversely the distance of the separated centrosomes at NEBD did not show a similar correlation (Figure 7C). To address the relevance of these results in another non-cancerous cell line and analyze the long-term effects of interrupting the nesprin N2G/FHOD1 interaction, we generated RPE-1 cells with inducible expression of these GFP-tagged protein domains (Figure 7F). Induction of N2G-H and FHOD (1–339) prevented colony formation in these cells and caused a significant reduction in cell proliferation as judged by nuclei counting (Figures 7G and 7H). Cells that survived for 5 days following induction of expression of N2G-H and FHOD(1–339) displayed micronuclei and showed aberrant nuclear morphology, suggesting that disrupting the LINC/actin interaction causes severe chromosome instability (Figure 7I).

## Discussion

This study reveals a mechanism that regulates the position of separating centrosomes at NEBD. Previous work has established that a symmetrical position of the centrosomes at NEBD (i.e., alignment along the nuclear diameter) is critical for the accurate establishment of sister chromatid biorientation (Kaseda et al., 2012, Silkworth et al., 2012). However, it remains unclear how the cells correlate cortical and nuclear geometry with the movement of the separating centrosomes prior to NEBD. Our assay of centrosome separation in 1NM-PP1-arrested cdk1as cells (Figure 1) supports the idea that a steady state position is reached at NEBD following Eg5 activation and that this stable position depends on the equilibrium of Eg5 and Eg5-antagonizing forces. Under physiological conditions, when Cdk1 activation is not prevented, we observed a doubling in the speed of separation and a decrease in the number of cells that reached optimal centrosome alignment at NEBD. Thus, the balance between fast Cdk1-driven centrosome separation and accurate positioning varies significantly in the cell population. Overall, it may be preferable to initiate centrosome separation early to allow time for optimal positioning, as suggested previously (Mardin et al., 2013).

A major contributor to this control mechanism is dynein acting both from the cell cortex and the NE. Our data in Figure 2 suggest that NE-associated dynein is the main contributor to centrosome positioning in G2 phase. First, dynein motor depletion and depletion of proteins that link dynein to the NE show almost identical phenotypes (Figure 2A). Second, the overall displacement of centrosomes induced by Eg5 inhibition is markedly increased, and directed movement in the cytoplasm occurs at a similar speed than in controls (Figures 2B–2D). Third, the effects of dynein depletion are compensated by co-depletion of kinesin-1 (Figure S2B). Thus, the balance of kinesin-1- and dynein-dependent pushing and pulling is critical to generate a dynamic association of the centrosomes to the NE, but not strictly required to generate a force that antagonizes Eg5-driven separation.

Our results emphasize that both MT polymerization and actin polymerization contribute significantly to the Eg5-antagonizing forces (Figures 2A, 3, and 4). A simple mechanism to explain force generation could be a MT-polymerization-dependent backward push at the actin barrier. Similar pushing forces have been proposed to contribute to centrosome centering and the generation of inward-directed pressure from astral MTs in the mitotic spindle (Burakov et al., 2003, Letort et al., 2016, van Heesbeen et al., 2017). However, we also observed considerable cross-talk between actin and MT structures and dynamics that further complicates the interpretation of our results. Further work will be required to dissect the precise mechanism that generates the forces that counteract Eg5 in prophase.

Strikingly, both Eg5-driven outward motion and inward motion driven by MT polymerization were asymmetric, with one centrosome moving more than the other. We did not detect a correlation between mobility and centrosome age, as previously suggested for centrosome mobility in G1 and early S phase (Piel et al., 2000) and during separation in asymmetric stem cell divisions (Yamashita and Fuller, 2008). Thus, other potentially stochastic events may generate differences in force exerted on the centrosome pair during separation.

Surprisingly, we found that centrosomes mostly moved toward a central position underneath the flat nucleus (Figure 4). Indeed, our analysis of cell-cycle-dependent centrosome position in U2OS and RPE-1 cells (Figure 4E) suggests that centrosomes preferentially reside in this position close to the nuclear centroid in G2 phase, while they are located close to the border of the NE in G1/S phase. When initiating centrosome separation from the nuclear centroid, the centrosomes always move in a radial fashion and remain in a symmetrical position. This mechanism may thus help to ensure the coordination of centrosome separation with regards to the nucleus prior NEBD.

Our observation of G2/prophase-specific perinuclear actin structures (Figure 5) suggests a mechanism for actin-dependent coordination of centrosome movement toward the nuclear centroid. A transient perinuclear actin ring has been observed previously (Münter et al., 2006) and appears to be triggered by Ca^2+^ signaling in response to mechanical stress (Shao et al., 2015). Recently, Booth et al. reported a similar prophase-specific actin structure in U2OS cdk1as and RPE-1 cells and implicated it in maintaining the positioning of condensed chromosomes at NEBD (Booth et al., 2019). This actin structure could, thus, have a wider function in supporting spindle formation and sister chromatid capture in prophase and early prometaphase. In contrast to our study, Booth et al. expressed a dominant-negative Klarsicht, ANC-1, Syne Homology (KASH) domain (Luxton et al., 2010, Starr and Han, 2002) that did not appear to impact on centrosome separation. This difference may be due to a stronger and more direct effect of dominant negative FHOD1 and nesprin N2G on the interaction with F-actin compared to the disruption of the KASH/Sad1p, UNC-84 (SUN)-domain interaction in the periplasmic space. The differential effects of FHOD1(1–339) and N2G-H expression on MT dynamics (Figures 6I and 6J) also point to a more complex and varied interplay in these structures with nuclear positioning and MT polymerization.

Disrupting the interaction of nesprin and F-actin prevented the formation of the perinuclear actin ring and had a significant impact on Eg5-antagonizing forces and the coordination between centrosome and nuclear position (Figure 6). Our model suggests that removing this barrier should cause an increase in centrosome distance at NEBD due to a reduction of Eg5-antagonizing forces. When centrosomes separated in cells that expressed these dominant-negative domains, they did indeed show a positioning defect and often lost contact with the NE. In asynchronous conditions, this correlated with a significant increase in chromosomes bridges in anaphase (Figure 7). However, we also found that centrosome separation itself was significantly impaired. Recent reports have documented actin polymerization at the centrosome (Obino et al., 2016), and F-actin has previously been implicated to support centrosome separation (Cao et al., 2010, Rosenblatt et al., 2004, Uzbekov et al., 2002). Moreover, expression of these fragments could also affect dynein and/or Eg5 localization and activity. Thus, NE-associated F-actin may play multiple roles in coordinating centrosome positioning and also supporting centrosome separation. This is further supported by the dramatic effects that induced expression of these domains exert on proliferation and genomic stability of RPE-1 cells (Figures 7F–7I). These data indeed suggest a critical role for the coordination of actin with the NE for cell survival and the maintenance of a stable and intact nucleus. Centrosome positioning defects may well contribute to these phenotypes, but other effects of these proteins on chromosome positioning or unrelated areas of nuclear dynamics are also likely to contribute.

Overall, our results highlight how centrosomes are subjected to geometrical cues from the nucleus to guide their positioning from the onset of separation. Both MT/F-actin interaction and NE-associated dynein play a critical role in this positional control network. Kinesin-14 motors such as KIF3C are also expected to contribute to this balance of forces, as recently demonstrated (Hata et al., 2019), and will be important to unravel further cross-talk between these mechanisms. Moreover, if a cell fails to establish accurate centrosome position at NEBD, spindle positioning pathways will continue to monitor the orientation and length of the spindle (Corrigan et al., 2015, Dunsch et al., 2012, Kiyomitsu and Cheeseman, 2013, Kwon et al., 2015, Zulkipli et al., 2018), increasing the robustness of this system. These mechanisms are likely to vary significantly between cell types and may be altered in tumor cells with amplified centrosomes and increased chromosome instability. A precise quantitative model of centrosome separation will be important to help analyze these differences and predict how these differences can be exploited therapeutically.

## STAR★Methods

### Key Resources Table

**Table:** 

REAGENT or RESOURCE	SOURCE	IDENTIFIER
**Antibodies**		

Monoclonal anti alpha-tubulin	ABCAM	ab7921, RRID:AB_2241126
Polyclonal anti rabbit CenpF	ABCAM	ab5, RRID:AB_304721
Polyclonal anti rabbit PCNT	Santa Cruz	sc**-**68929; RRID:AB_2252070
Monoclonal anti mouse DHC	Santa Cruz	sc-514579; RRID n.a.
Monoclonal anti mouse DIC1	Abcam	ab23905; RRID:AB_2096669
Polyclonal anti rabbit Kinesin-1 (Kif5B)	Bethyl	A304-306A; RRID:AB_2620502
Polyclonal anti rabbit MCAK	Abcam	Ab228016; RRID n.a.
Polyclonal anti rabbit chTog	Abcam	Ab236981; RRID n.a.
Monoclonal anti rabbit GAPDH (6C5)	Abcam	Ab8245/ lot GR3185172-3; RRID:AB_2107448
Donkey anti rabbit Alexa Fluor 647	Invitrogen	A31573/ lot 1903516; RRID:AB_2536183
Donkey anti mouse Alexa Fluor 488	Invitrogen	A21202/ lot 1820538; RRID:AB_141607
Donkey anti goat Alexa Flour 594	Invitrogen	A11058/ lot 440197; RRID:AB_2534105
Goat anti rabbit immunoglobulins	Dako	P0448/ lot 20047670; RRID:AB_2617138
Goat anti mouse immunoglobulins	Dako	P0447/ lot 20030309; RRID:AB_2617137

**Chemicals and Inhibitors**		

1NMPP1	Calbiochem	529581
Nocodazole	Sigma	M1401
Cytochalasin	Sigma	C8273
Blebbistatin	Sigma	203390
Phalloidin	Sigma	P1951/49409
STLC ((+)-S-Trityl-L-cysteine	Sigma	164739
SiR Actin	Spirochrome	SC001
SiR Tubulin	Spirochrome	SC002
Experimental Models: Cell Lines		
U2OS	ATCC	HTB-96
RPE-1	ATCC	CRL-4000
MCF10A	ATCC	CRL-10317
HCT116	ATCC	CCL-247
BJ4	ATCC	CRL-2522
48BR	Penny Jeggo	N/A

**Recombinant DNA**		

GFP-FHOD1(1-339 expression vector	Greg Gundersen	N/A
GFP-N2G-H expression vector	Greg Gundersen	N/A
Sleeping Beauty inducible expression vector	Addgene	60496
Sleeping Beauty 100x Transposase	Addgene	34879

**siRNA**		

DHC	Dharmacon	OnTargetPlus

**DIC Dharmacon OnTargetPlus**		

Kinesin-1	Dharmacon	OnTargetPlus
CENPF	Dharmacon	OnTargetPlus
Asunder	Dharmacon	OnTargetPlus
MCAK	Dharmacon	OnTargetPlus
chTog	Dharmacon	OnTargetPlus

**Software**		

ImageJ 1.51h	ImageJ	N/A
Python 2.7	Anaconda	N/A
Segmentation and quantification of Actin Ring	Python	https://github.com/fabio-echegaray/contour-field
Cell and Nuclear Segmentation and centrosome tracking	Python	https://github.com/fabio-echegaray/centrosome-tracking

### Resource Availability

#### Lead Contact

Further information and requests for resources and reagents should be directed to and will be fulfilled by the Lead Contact, Helfrid Hochegger (hh65@sussex.ac.uk)

#### Materials Availability

Novel cell lines and plasmids described in this study are available on request

#### Data and Code Availability

Original source code for image segmentation and analysis is available at https://github.com/fabio-echegaray/contour-field and https://github.com/fabio-echegaray/centrosome-tracking.

### Experimental Model and Subject Details

U2OS, RPE-1 and HCT116 cells were cultured in Dulbecco’s modified Eagle Medium supplemented with 10% FCS, 2 mM L-glutamine, 100 U/ml penicillin and 0.1 mg/ml streptomycin. MCF10A were cultured in Dulbecco’s modified Eagle Medium F12 medium supplemented with 5% final horse serum, 20 ng/mL EGF, 0.5mg/ml Hydrocortisone, 100 ng/mL Cholera Toxin, 10μg/ml Insulin and 100 U/ml penicillin and 0.1 mg/ml streptomycin. BJ4 and 48BR cells were cultured in MEM supplemented with 10%FCS and 100 U/ml penicillin and 0.1 mg/ml streptomycin. All cells were regularily tested for mycoplasma infection and underwent identify verification by the GDSC tissue cukture facility. Cells were cultured at 37°C, in a 5% CO2 incubator.

### Method Details

#### Expression constructs and stable cell line generation

Stable U2OS cells were established expressing mEmerald-alpha-Tubulin (M. Davidsson, Addgene 54292) and RFP-PACT domain of pericentrin (a gift from Viji Draviam), mApple-EB3-7 (Addgene 54892, M. Davidson), or pFusionRed-H2B (Evrogen) using G418 selection. Stable U2OS cells were also established expressing RFP-PACT then EGFP-cenexin after lentiviral infection (pLXV-EGFP-C3-cenexin from addgene #73334, M. Thery). GFP-Nesprin N2G-H and FHOD1(1-339) expression vectors were a gift from Greg Gundersen (Kutscheidt et al., 2014).

TET-on Sleeping beauty plasmid (Kowarz et al., 2015) was obtained from Addgene (plasmid nr. 60496 pSB-tet-BP) with a Blue Fluorescent Protein (BFP) selection marker. The plasmid originally contains Luciferase which was replaced by the ORF of GFP, GFP-FHOD1(1-339) or GFP-N2G-H using PCR and NEB HiFi Assembly. We used BspDI and NcoI sites to cut out the luciferase and incorporated our GOI. 1.9⎧g of this plasmid along with 100ng transposase enzyme SB-100X (Addgene plasmid nr. 34879) was transfected into RPE1 degron cells using electroporation. Afterward, cells were grown for 10 days and FACS sorted into a 96-well plate for BFP expression (excitation approx. 456nm) using FACSMelody sorter according to the manufacturer instructions. Cells were then grown up and analyzed for protein expression after Doxycycline addition using immunoblotting.

#### Antibodies

Primary antibodies were used at manufacturer’s recommended concentrations and are listed in the table below.

#### siRNA transfections

Cells were seeded in six-well plates at a density of 0.4 × 105 cells/ml and were reverse transfected with 20 nM siRNA using Lipofectamine RNAiMax transfection reagent (Invitrogen) following the manufacturers’ protocol; 3 days later cells were then prepared for live cell imaging or western blotting as described. SMARTpool ON-Target plus were purchased from Dharmacon

#### Immunofluorescence and live cell microscopy

U2OS and RPE cells were grown on coverslips and fixed for 10 min in 3.7% formaldehyde, rinsed 3 times in PBS. Coverslips were then rinsed in PBS and cells permeabilized in PBS-0.1% NP40. Cells were blocked in 2% BSA for 10 min and probed with primary antibodies (as indicated in figure legends) for 40 min. Slides/coverslips were rinsed 4 × in PBS and probed with Alexa secondary antibodies listed for 20 min. Slides/coverslips were then rinsed 4 × in PBS and coverslips were mounted using ProLong® Gold mounting solution containing DAPI (Invitrogen). For image acquisition, we used a Olymnpus IX81 microscope equipped for spinning disk confocal microscopy (Yokogawa disk, CSU-X M1) by 3i (Intelligent Imaging Innovations). Imaging was performed using a UPLanS Apo, N.A. 1.35, × 60 oil immersion objective (Olympus), standard filter sets (excitation 360/40; 490/20; 555/28; emission 457/50; 528/38; 617/40) and a Evolve PVCAM camera (Photometrics). Z-series of 0.7 μm stacks were acquired using Slidebook software (Version 6.0.8) and images exported as tiff files. Time-lapse microscopy was performed on glass bottom 35mm dishes from MatTech (P35G-1.5-14C), or IBIDI μ-slides Grid-500 in CO2-independent medium (Invitrogen) in an environmental chamber (Digital Pixels) heated to 37°C. A total of 7.7 μM stacks were taken at 5 min intervals unless otherwise indicated. Maximum intensity projections of the time series were exported into mp4 format for presentation as Supplementary Videos.

#### Image segmentation, tracking and analysis

Images were analyzed in ImageJ. Regions were manually marked on the image for analysis. The image was smoothed with a difference of Gaussians filter using radii above and below the expected feature size (1.5 and 4.5 pixels for spots of size 3). Centrosomes were identified using the FindFoci algorithm (Herbert et al., 2014) with Otsu thresholding (Otsu, 1979) to define the background and a minimum spot size of 15 pixels above the background. Spots were discarded, if the calculated circularity was less than 0.7, i.e., elongated spots. For centrosome separation and distance analysis centrosomes were tracked in 3D using the Spot Distance ImageJ plugin (http://www.sussex.ac.uk/gdsc/intranet/microscopy/UserSupport/AnalysisProtocol/imagej/plugins/) with tracking function to obtain the (x, y, z) position of both centrosomes. The positions of the centrosomes were imported to MATLAB(R) where they were further analyzed. Tracks were generated and visualized with the MATLAB(R) package “Phagosight” (Henry et al., 2013). For validation, each pair of tracks was displayed and visualized with different colors and markers. Numerous measurements were extracted from the tracks: number of time points of the tracks, time of first contact, distance of centrosome separation, individual centrosome velocity, approximation velocity, final separation, and mean squared displacement. Bootstrapped confidence intervals were calculated using the Python Seaborn plotting library (https://seaborn.pydata.org/index.html). For statistical analysis p values comparing two sets of single cell data were calculated using a Student’s test.

To track centrosomes movement relative to the nuclear centroid, we segmented the nucleus and centrosomes based on Hoechst 33342 and RFP-PACT staining. We computed the centroid of the nuclear masks in each image and referenced the centrosome position relative to that point using vector algebra. Tracking was performed using the TrackMate plugin from ImageJ. For RFP channel -centrosome position- tracking, we detected particles using the Laplacian of Gaussian (LoG) transform, tracked them using a Kalman Filter, and finally extracted candidates using track lengths and gaps criteria. For the UV channel -nuclei- tracking we applied an intensity threshold to the image and morphological close and open operations to remove holes and noise respectively. We applied a distance transform and Gaussian blur and then used watershed transform to cut merged nuclei. After these steps, the nuclear masks were tracked in the same way as centrosomes in the RFP channel. The cell shape was segmented using the GFP-Tubulin channel pre-processed with a Gabor Filter. Centrosome distance relative to centroid was calculated using vector addition. Eb3 cap tracking and quantification was performed on Eb3-cherry channel using Trackpy (https://soft-matter.github.io/trackpy/v0.3.2/) after subtracting the first image to the entire sequence. Drift prediction and nearest velocity with a search radius of 3 pixels were used as prediction algorithms in Figures 3 and S3. Manual tracking was performed to quantify EB3 comet moving in Figure 6.

### Quantification and Statistical Analysis

All experiments included at least three independent biological repeats. Sample size per repeat varied between experiments and are indicated in the Figure Legends. Sample size was based on standard practise in cell biological assays and not specifically pre-estimated. p values were calculated using an independent two sample t test. Levels of significance are indicated by stars (^∗^ p < 0.05, ^∗∗^ p < 0.01, **^∗∗∗^**p < 0.001). For all experiments, samples were not randomized and the investigators were not blinded to the group allocation during experiments and outcome assessment. No exclusion criteria were used and all collected data were used for statistical analysis.

## Supplemental Information

Document S1. Figures S1–S5

Document S2. Article plus Supplemental Information
