# Supplementary material for: Prophase-Specific Perinuclear Actin Coordinates Centrosome Separation and Positioning to Ensure Accurate Chromosome Segregation
Source: Cell Rep. 2020 May 26;31(8):107681. doi: 10.1016/j.celrep.2020.107681 (PMC7262599; doi:10.1016/j.celrep.2020.107681)
Supplement: Document S1. Figures S1–S5 [file mmc1.pdf]

Cell Reports, Volume *31*

Supplemental Information

Prophase-Specific Perinuclear Actin Coordinates Centrosome Separation and Positioning to Ensure Accurate Chromosome Segregation

Tom Stiff, Fabio R. Echegaray-Iturra, Harry J. Pink, Alex Herbert, Constantino Carlos Reyes-Aldasoro, and Helfrid Hochegger

Supplemental Information

Prophase-specific perinuclear Actin coordinates centrosome separation and positioning to ensure accurate chromosome segregation

Tom Stiff, Fabio R. Echegaray-Iturra, Harry J. Pink, Alex Herbert, Constantino C. Reyes-Aldasoro, Helfrid Hochegger

Figure S1

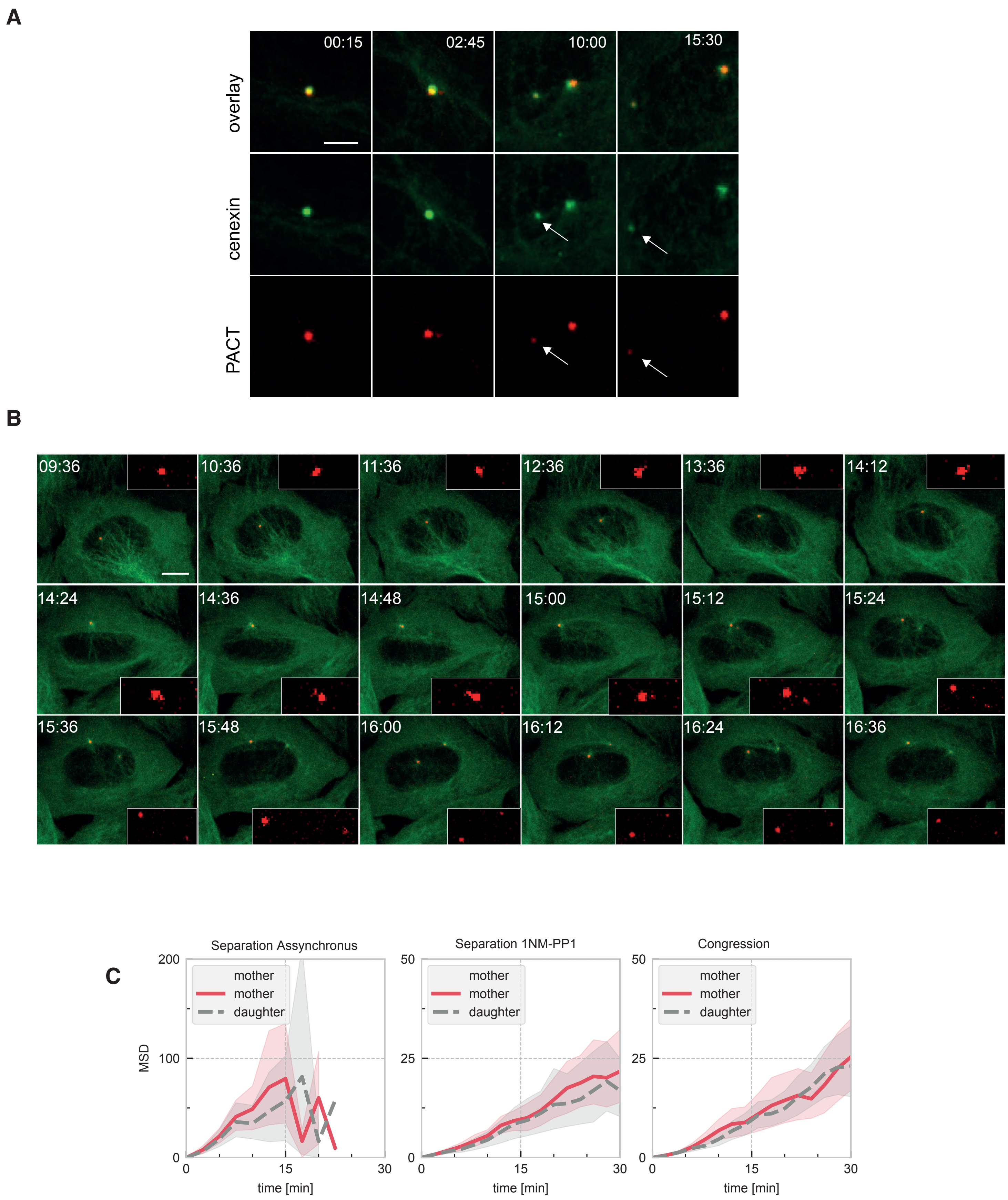
A
00:15
02:45
10:00
15:30
overlay
cenexin
PACT
B
09:36
10:36
11:36
12:36
13:36
14:12
14:24
14:36
14:48
15:00
15:12
15:24
15:36
15:48
16:00
16:12
16:24
16:36
C
Separation Assynchronus
Separation 1NM-PP1
Congression
MSD
mother
mother
daughter
time [min]

S1 (relating to Figure 1) Lack of correlation between centrosome mobility and age during separation/congression

A) Correlation of centrosome age and GFP-Cenexin/RFP-PACT expression by live cell imaging. Shown are stills from a live cell experiment (time is indicated as hh:min) where a G1 cell was followed through S-phase. The younger centrosome
can be recognised by a lower signal of GFP-Cenexin (green), that is mirrored by a lower RFP-PACT (red) signal, (scalebar=10μm).

B) Identification of mother/daughter centrosome based on RFP-PACT staining. Cells were treated with 1NMPP1 and centrosome duplication and separation was followed by live cell imaging (green: GFP-Tubulin; red: RFP-PACT, scalebar=10μm). The centrosome with the lower amount of RFP-PACT was classified as the younger centrosome. Following this classification we performed tracking and MSD analysis.

C) MSD analysis of mother and daughter centrosomes. Centrosomes were classified by the PACT signal as mother and daughter, and MSD of the individual tracks was analysed for centrosome separation in asynchronous cells, following STLC wash-out in 1NM-PP1 arrested cells and during congression. The bold lines show the

Figure S2

A

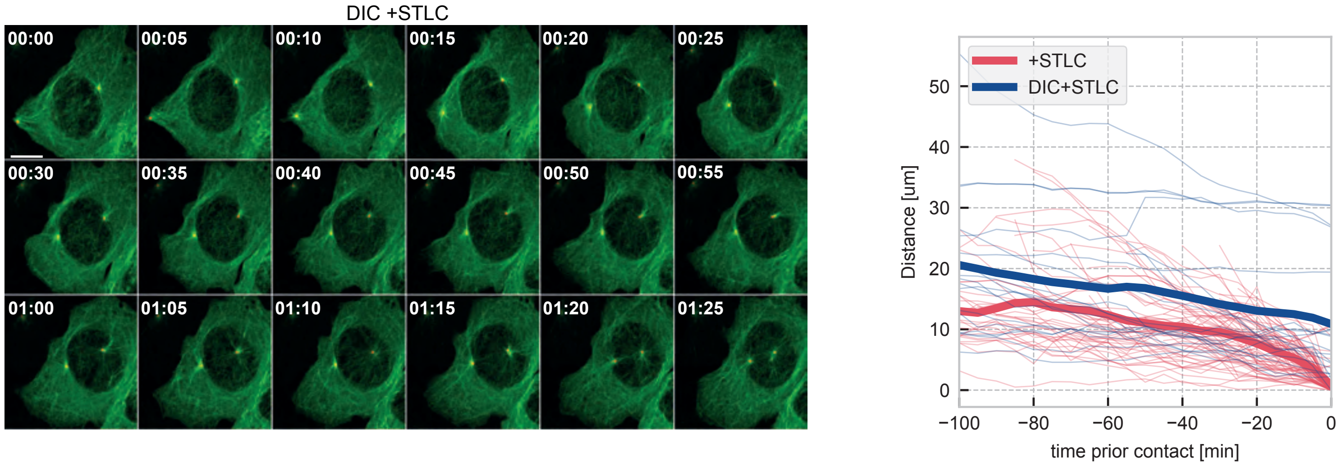

B

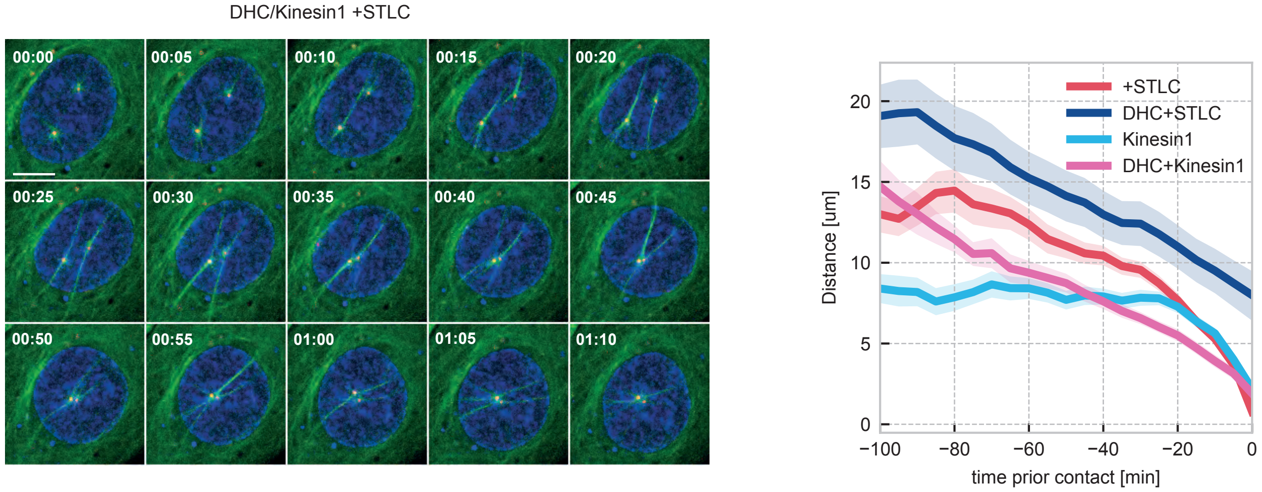

C

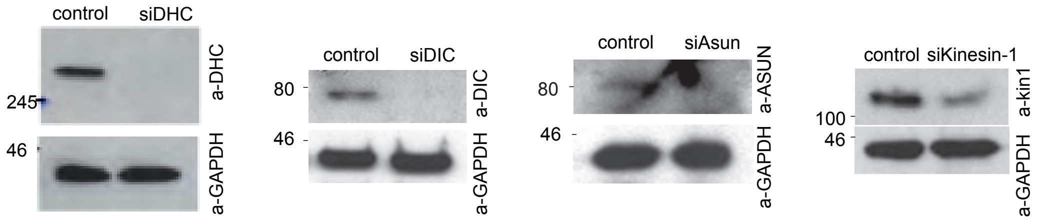

S2 (relating to Figure 2) Effects of DIC and Dynein/Kinesin-1 depletion on centrosome congression

A) Analysis centrosome congression in a DIC depleted cell. Left panel shows an imaging time course of a representative DIC depleted cell U2OS cdk1as cells following STLC treatment. Time is indicted in hh:min, scale bar represent 10µm. Right Panel shows individual distance/time tracks comparing control (red) with DIC depleted (blue) cells. Bold lines show the mean distance for each condition (for each track n>25).
B) Left Panel: Images from time-lapse video of centrosome congression in DHC + Kinesis-1 depleted cells after STLC treatment Time is indicated in hh:min on the top right, scale bar represent 10µm). Right Panel: distance/time tracks comparing control (red) with cells transfected with indicated siRNAs. Bold lines show the mean distance for each condition (for each track n>25).
C) Immuno-blots of cells transfected with indicated siRNAs, probed with indicated antibodies.

Figure S3

A

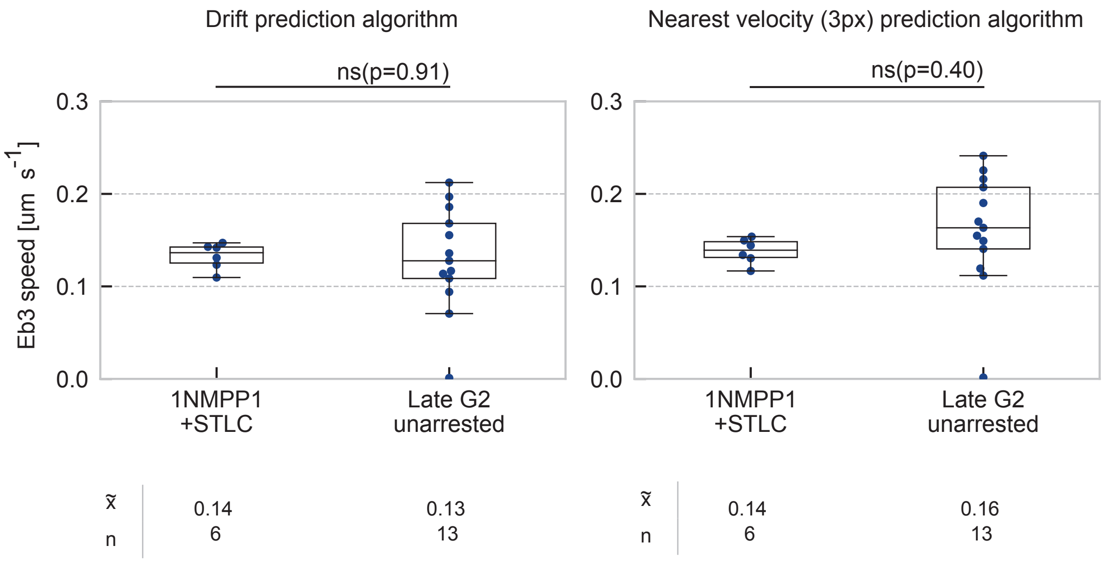
Drift prediction algorithm
Nearest velocity (3px) prediction algorithm
ns(p=0.91)
ns(p=0.40)
Eb3 speed [um s^{-1}]
0.3
0.2
0.1
0.0
1NMPP1
+STLC
Late G2
unarrested
x̃
n
0.14
6
0.13
13
0.14
6
0.16
13

B

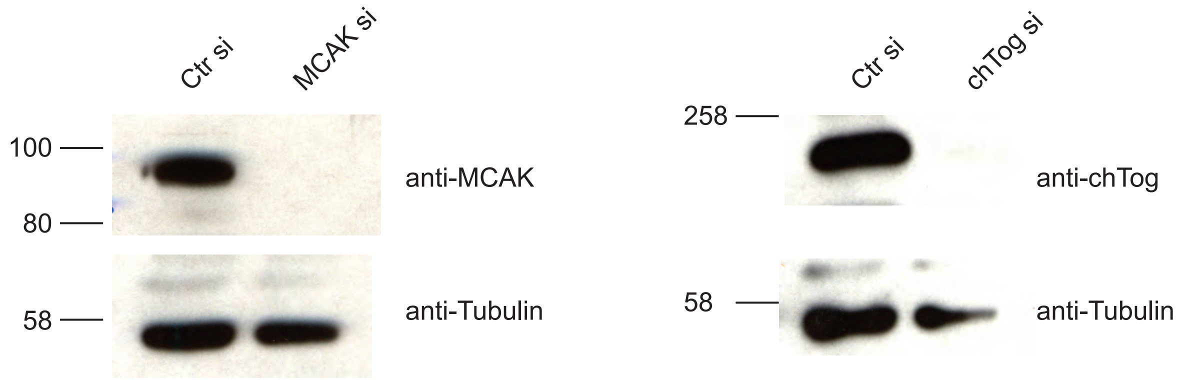
Ctr si
MCAK si
100
80
58
anti-MCAK
anti-Tubulin
Ctr si
chTog si
258
58
anti-chTog
anti-Tubulin

S3 (relating to Figure 3) EB3 tracking in 1NM-PP1 arrested and asynchronous cells

A) Quantification of MT tracking in RFP-EB3 expressing cells using drift prediction and nearest velocity tracking
algorithms (see material and methods section). Cells were either arrested in 1NMPP1, or cells with intact NE and
separated centrosomes were picked for analysis in asynchronous populations. Median values per cells (>10 tracks over cells) are shown. (x) indicates overall median speed, and n= the number of cells analysed.
B) Immuno-blots of cells transfected with indicated siRNAs, probed with indicated antibodies.

Figure S4

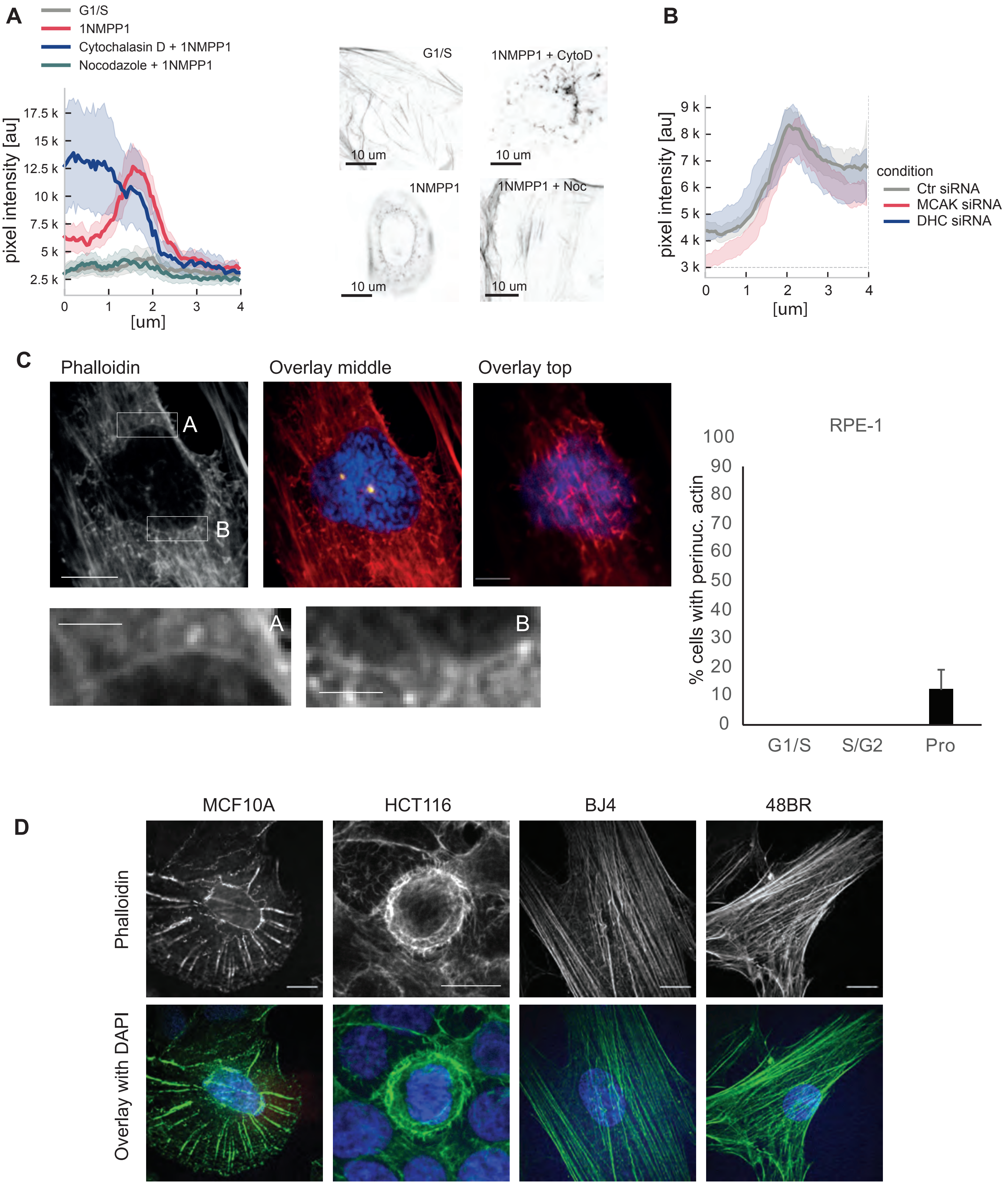
A
G1/S
1NMPP1
Cytochalasin D + 1NMPP1
Nocodazole + 1NMPP1
pixel intensity [au]
17.5 k
15 k
12.5 k
10 k
7.5 k
5 k
2.5 k
0
1
2
3
4
[um]
G1/S
1NMPP1 + CytoD
1NMPP1
1NMPP1 + Noc
10 um
B
pixel intensity [au]
9 k
8 k
7 k
6 k
5 k
4 k
3 k
[um]
condition
Ctr siRNA
MCAK siRNA
DHC siRNA
C
Phalloidin
Overlay middle
Overlay top
A
B
RPE-1
% cells with perinuc. actin
100
90
80
70
60
50
40
30
20
10
0
G1/S
S/G2
Pro
D
MCF10A
HCT116
BJ4
48BR
Phalloidin
Overlay with DAPI

S4 (relating to Figure 5) Prophase specific peri-nuclear Actin

A) Quantification of perinuclear actin formation in asynchronous and 1NM-PP1 arrested U2OS cdk1as cells after Cytochalasin D (2µg/ml) or Nocodoazole (20ng/ml) treatment. These additional treatments were added 2 hours prior to fixation.
Quantification of line intensity across randomly selected sections across the NE are shown in the left panel. The images show phalloidin staining in representative cells, **B)** Quantification of perinuclear actin (as in A) formation in 1NMPP1 arrested U2OS cdk1as cells 48 hours after transfection with indicated siRNAs. **C)** Images of fixed RPE-1 cells probed by immunofluorescence and phalloidin staining. Left panels show images of phalloidin stained single stack (scale bar =10µm), and magnified areas are displayed below (scale bar = 2.5µm). The middle panels show maximum intensity projections of 4 stacks covering the middle layer of the cells, the overlay shows Phalloidin (red), alpha-tubulin (green) and DAPI (blue), the right panels show projections of 4 layers at the top of the cell. Scale bar is 10µm for large panels and 2.5 µm for zoomed panels. Graphs on the right show average percentage of cells in which we detected perinuclear actin structures in indicated cell cycle phases. **D)** Perinuclear actin in prophase MCF10A, HCT116, BJ4 and 48BR cells. Images of representative cells stained with phalloidin (top panels) and co-labelled with DAPI (bottom panel), the scale bar represents 10µm. Note the presence of abundant stress fibres in the primary cell lines (BJ4 and 48BR). The Prophase state was identified by partial chromosome condensation.

Figure S5

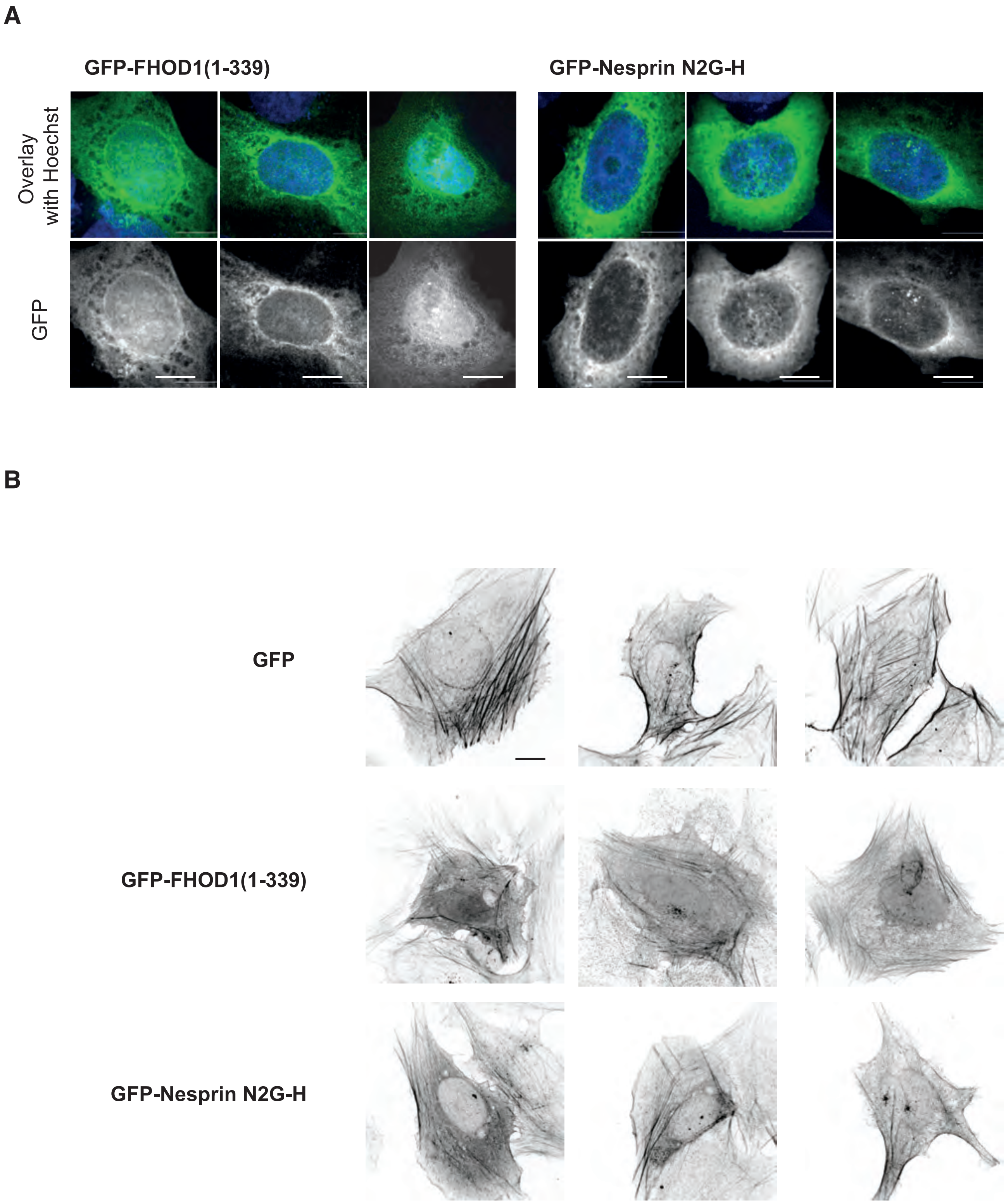
A
GFP-FHOD1(1-339)
GFP-Nesprin N2G-H
Overlay with Hoechst
GFP
B
GFP
GFP-FHOD1(1-339)
GFP-Nesprin N2G-H

S5 (relating to Figure 6) GFP-FHOD1(1-339) and GFP-N2G-H expression in 1NM-PP1 arrested U2OS cdk1as cells

A) Top panels show overlay of GFP (green) and Hoechst (blue), bottom panel shows images from GFP channel
in B/W look up table. The scale bar indicates 10μm.
B) Examples of phalloidin stained 1NMPP1 arrested cdk1as cells following transient expression of indicated proteins. The scale bar indicates 10μm.
